# Spinal cord injury-induced gut dysbiosis influences neurological recovery partly through short-chain fatty acids

**DOI:** 10.1038/s41522-023-00466-5

**Published:** 2023-12-14

**Authors:** Yingli Jing, Degang Yang, Fan Bai, Qiuying Wang, Chao Zhang, Yitong Yan, Zihan Li, Yan Li, Zhiguo Chen, Jianjun Li, Yan Yu

**Affiliations:** 1 China Rehabilitation Science Institute, China Rehabilitation Research Center, Beijing Key Laboratory of Neural Injury and Rehabilitation, and School of Rehabilitation Medicine, Capital Medical University, Beijing, 100068 China; 2 Center of Neural Injury and Repair, Beijing Institute for Brain Disorders, Beijing, 100068 China; 3 Department of Spinal and Neural Function Reconstruction, Beijing Bo’ai Hospital, Beijing, 100068 China; 4 Department of Neurosurgery, Linyi People’s Hospital, Shangdong, 276034 China; 5 Cell Therapy Center, Beijing Institute of Geriatrics, Xuanwu Hospital Capital Medical University, National Clinical Research Center for Geriatric Diseases, and Key Laboratory of Neurodegenerative Diseases, Ministry of Education, Beijing, 100053 China

**Keywords:** Clinical microbiology, Health care

## Abstract

Spinal cord injury (SCI) can reshape gut microbial composition, significantly affecting clinical outcomes in SCI patients. However, mechanisms regarding gut–brain interactions and their clinical implications have not been elucidated. We hypothesized that short-chain fatty acids (SCFAs), intestinal microbial bioactive metabolites, may significantly affect the gut–brain axis and enhance functional recovery in a mouse model of SCI. We enrolled 59 SCI patients and 27 healthy control subjects and collected samples. Thereafter, gut microbiota and SCFAs were analyzed using 16 S rDNA sequencing and gas chromatography–mass spectrometry, respectively. We observed an increase in *Actinobacteriota* abundance and a decrease in *Firmicute*s abundance. Particularly, the SCFA-producing genera, such as *Faecalibacterium*, *Megamonas*, and *Agathobacte*r were significantly downregulated among SCI patients compared to healthy controls. Moreover, SCI induced downregulation of acetic acid (AA), propionic acid (PA), and butyric acid (BA) in the SCI group. Fecal SCFA contents were altered in SCI patients with different injury course and injury segments. Main SCFAs (AA, BA, and PA) were administered in combination to treat SCI mice. SCFA supplementation significantly improved locomotor recovery in SCI mice, enhanced neuronal survival, promoted axonal formation, reduced astrogliosis, and suppressed microglial activation. Furthermore, SCFA supplementation downregulated NF-κB signaling while upregulating neurotrophin-3 expression following SCI. Microbial sequencing and metabolomics analysis showed that SCI patients exhibited a lower level of certain SCFAs and related bacterial strains than healthy controls. SCFA supplementation can reduce inflammation and enhance nourishing elements, facilitating the restoration of neurological tissues and the improvement of functional recuperation. Trial registration: This study was registered in the China Clinical Trial Registry (www.chictr.org.cn) on February 13, 2017 (ChiCTR-RPC-17010621).

## Introduction

Spinal cord injury (SCI) is a destructive disease, with patients suffering from severe neurological impairments, including motor and/or sensory deficits, and autonomic dysfunction below the injury level. The disability caused by SCI has a significant impact on patient’s lives and increases the care and economic burden on families and society^1^. SCI is a lifelong nervous system disease because available treatments are restricted^1,2^. Recently, an increased understanding of the bidirectional communication of microbiota with the central nervous system (CNS), termed the microbiota–gut–brain axis, has opened new areas of investigation for SCI research, which may provide novel treatments of SCI.

Many studies have implicated changes or diversification in gut microbial composition along with SCI progression and in patients with different severity of SCI^3–8^. Gungor et al. showed that the gut microbial composition was altered following SCI; particularly, butyrate-producing bacterial abundance decreased among patients with SCI compared with normal controls^3^. Bazzocchi et al. reported that gut microbiota in SCI cases showed typical dysbiotic features, including an increased abundance of potentially pathogenic, pro-inflammatory, and mucus-degrading bacterial species and depleted short-chain fatty acid (SCFA) producer species, which is tightly associated with disease completeness and severity^5^. Furthermore, changing gut microbiota through fecal microbiota transplantation or administering probiotics or melatonin during experimental SCI could remarkably improve functional recovery^9–11^. SCI clinical studies^4–8,11,12^ and animal experiments^9–11^ showed that SCI can alter gut homeostasis, subsequently leading to gut dysbiosis, which exacerbates the detrimental effects of SCI^9–11^. Mechanism related to the gut–microbiota–CNS crosstalk has gained increasing attention. Numerous possible pathways related to the gut–microbiota’s influence on the CNS have been reported^12^. The microbiota and CNS can establish communication in both directions through pathways like the immune system, vagus nerve, hypothalamic–pituitary–adrenal (HPA) axis, tryptophan metabolism, and the enteric nervous system. This interaction could encompass specific neurotransmitters and microbial metabolites that play pivotal roles in influencing states of health and disease^13,14^. However, the mechanisms around those mediators have not been fully studied, and their significance remains to be elucidated in the context of SCI.

Our study focused on the metabolite groups of SCFAs, including acetate, butyrate, and propionate. These compounds represent major metabolites generated through non-digestible carbohydrate fermentation via gut bacteria, which can readily cross the blood–brain barrier and play a crucial immunomodulatory role in the CNS^15–18^. For example, administering SCFAs can improve functional recovery via immunological mechanisms in distal permanent occlusion of the middle cerebral artery model^19^. SCFA treatment retarded disease progression in experimental autoimmune encephalitis models by reducing axonal damage and inhibiting anti-inflammatory mechanisms^20^. Furthermore, addition of SCFA addition mitigates the repeated psychosocial stress-mediated selective and persistent negative effects^21^. SCFAs may have beneficial effects on neurodegenerative diseases and post-ischemic neurogenesis^22,23^.

So far, gut microbiome, fecal SCFAs, and the potential relationships with neurological recovery remain to be elucidated in patients with SCI and animal models. The current study aimed to explore the mechanism underlying SCI-induced gut dysbiosis and its influence on functional deficits following SCI. The results may shed light on a potential therapeutic use of bioactive metabolites in the microbiome for post-injury recovery of SCI patients.

## Results

### Patient and control groups

Table 1 shows the baseline characteristics of enrolled patients with SCI and healthy controls. Age and sex were not significantly different between the two groups.

**Table 1:** Demographic characteristics of SCI patients and healthy controls.

Variable	Patients with SCI (*n* = 59)	Healthy controls (*n* = 21)
Gender (male; female)	49;10	22;5
Age, year(mean ± SEM)	37.00 ± 1.40	40.63 ± 1.88
Time interval between lesion and fecal sampling		
<=3 months (mean ± SEM)	10 (2.10 ± 0.23)	–
<=6 months (mean ± SEM)	11 (5.18 ± 0.23)	–
<=12 months (mean ± SEM)	13 (9.62 ± 0.57)	–
>12 months (mean ± SEM)	25 (61.88 ± 10.57)	–
Neurologic lesion level		
C1-T5(%)	24 (40.68%)	–
T6-l5(%)	35 (59.32%)	–
Traumatic etiology (%)		
Motor vehicle collisions	28 (47.46%)	–
Fall from an elevated height	15 (25.42%)	–
Injured by falling object	16 (27.12%)	–

The disease course of enrolled patients is mostly distributed within 2 year after injury. The injury segment was mostly found at T10, followed by T1, in patients with SCI. All patients with SCI had neurologically complete injuries (ASIA grade A), resulting from spinal cord contusion. Etiology analysis showed that traffic accidents accounted for approximately 50% of traumatic spinal cord injuries. Furthermore, healthy participants were recruited from a qualified population in the same hospital, including nursing staff or accompanying relatives. All participants did not report another disease.

### Patients with SCI have altered gut microbiota profiles and SCFA contents

We characterized the gut microbiota profiles by analyzing a total of 82 fecal samples from SCI patients and normal controls. Rarefaction curves showed that sequencing depth covered a majority of diversity and rare new phylotypes (Fig. S1a). A decrease was observed in both the Chao index and Shannon index following the injury; however, the observed differences were not statistically significant (Fig. S2). Weighted UniFrac PCoA analysis demonstrated distinctive clustering of the SCI group in comparison to the control group (Fig. S1b). The SCI group exhibited distinct microbial profiles at both the phylum and genus levels when compared to the control group (Figs. S3, S1c, and S1d). A cladogram was plotted showing the gut microbial structure, dominant bacteria, and taxonomic differences between the two groups. Figure S1e, f shows significant differences in genera. Compared with the control group, the SCI group had reduced *Faecalibacterium*, *Megamonas*, and *Agathobacter* relative abundances; additionally, the SCI group had increased *Enterococcus* and *Klebsiella* abundances. We proceeded to analyze the metabolic function profile using a metagenomic approach (PICRUSt2)^24^. The outcomes indicated that the injury potentially led to a reduction in fecal SCFAs due to a reduced abundance of enzymes necessary for SCFAs synthesis (Fig. S4).

**Fig. 1:**
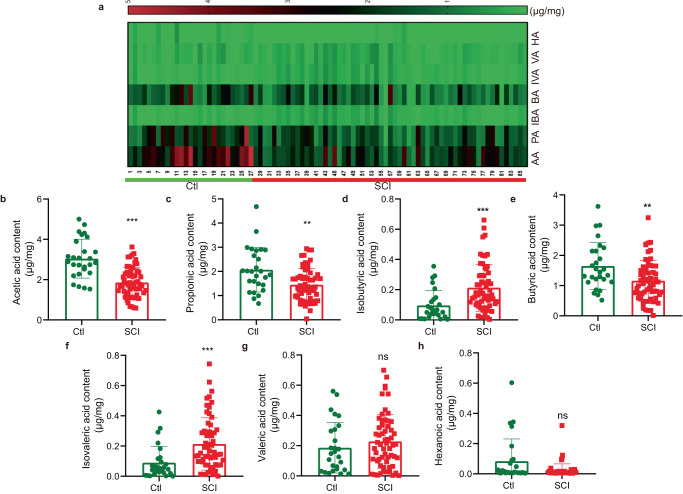
Aberrant SCFA levels in patients with SCI. **a** The heatmap showing fecal SCFA levels between the control and SCI groups. Quantitative analyses of SCFA levels, including acetic acid (AA) (**b**), propionic acid (PA) (**c**), isobutyric acid (IBA) (**d**), butyric acid (BA) (**e**), isovaleric acid (IVA) (**f**), valeric acid (VA) (**g**), and hexanoic acid (HA) (**h**) were performed by using a gas chromatography–mass spectrometry (GC-MS) between the control and SCI groups. Two-tailed paired *t* tests were used for comparisons between two groups. SEM were used to represent error bars. *p < 0.05; **p < 0.01, ns not significant.

The gut microbiota in patients with SCI showed a dysbiotic signature featuring reduced SCFA levels. Our previous study showed that SCFA levels were altered following SCI in a mouse model^9^. However, whether SCFA levels also change in patients with SCI remains unclear. GC-MS was used to measure SCFA contents, such as acetic acid (AA), butyric acid (BA), hexanoic acid (HA), propionic acid (PA), valeric acid (VA), isobutyric acid (IBA), and isovaleric acid (IVA) (Fig. 1). Compared with the control group, SCI induced AA, PA, and BA downregulation (Fig. 1b, c, e). In contrast, IBA and IVA levels were higher in the SCI group than in the control group (Fig. 1d, f). Moreover, VA and HA contents were not significantly different between the two groups (Fig. 1 g, h). Compared with the control group, the SCI group showed that the AA level in examined fecal SCFAs was most significantly changed, decreasing by 40.14%. Similar to AA, PA and BA levels in SCI mice were reduced by 30.03% and 29.57%, respectively (Fig. 1a–c). The levels of circulating SCFAs were also measured. The extent of change in circulating SCFAs in both groups was not as substantial as that observed in stool samples. In comparison to the control group, a significant difference was only observed in the concentration of BA but not in the concentration of AA and PA between the two groups (Fig. S5). The analysis was based on 21 blood samples from control subjects and 13 blood samples from patients. Future studies will be conducted to expand the cohort size.

### Fecal SCFA levels are altered in SCI patients with different injury course

**Fig. 2:**
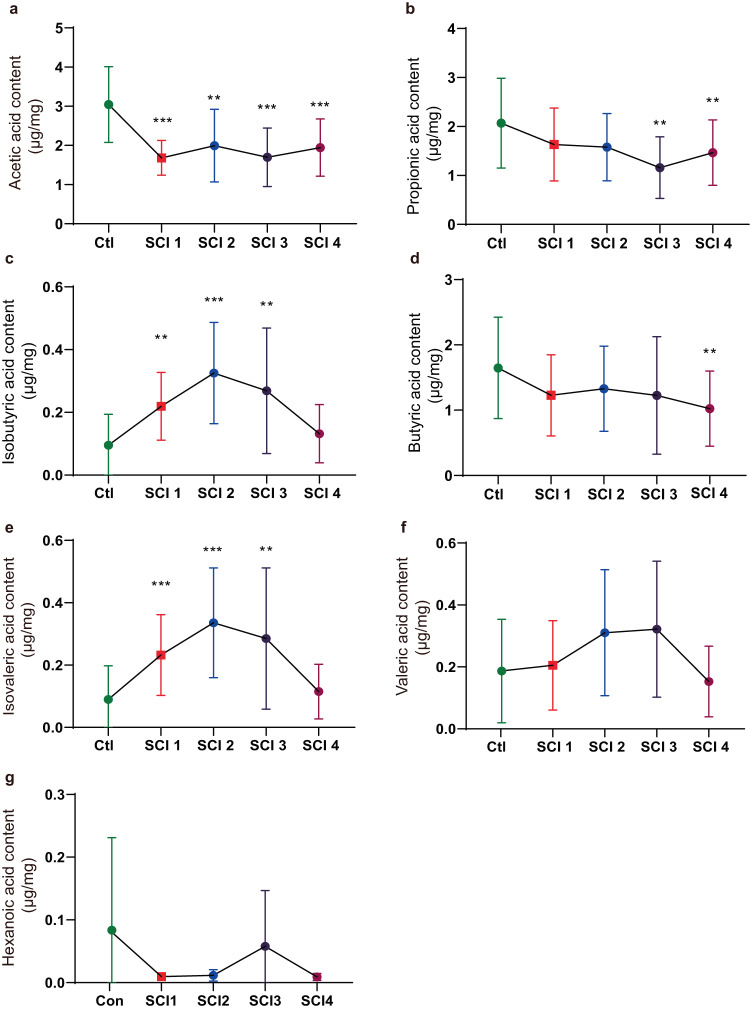
Aberrant SCFA levels in patients with SCI with different injury course. Quantitative analyses of SCFA levels, including AA (**a**), PA (**b**), IBA (**c**), BA (**d**), IVA (**e**), IVA (**f**), and HA (**g**), were performed by using GC-MS among different groups. One-way ANOVA followed by Tukey’s post-hoc test was used for comparisons among multiple groups and two-tailed paired *t* tests were used for comparisons between two groups. SEM were used to represent error bars. *p < 0.05; **p < 0.01, ns not significant (SCI 1, within 3 months following SCI; SCI 2, within 3–6 months following SCI; SCI 3, within 6–12 months following SCI; SCI 4, over 12 months following SCI).

The SCFA levels in patients with SCI were significantly decreased compared with the control group. To examine whether SCFA levels changed with the duration and progression of SCI, the enrolled patients were further stratified into subgroups: SCI 1, within 3 months following SCI; SCI 2, 3–6 months following SCI; SCI 3, 6–12 months following SCI; and SCI 4, over 12 months post-injury. Compared with the control group, the AA level was significantly reduced in the SCI 1 to SCI 4 groups (Fig. 2a). The PA level exhibited a declining pattern in the subgroups of individuals with SCI, and this decline reached statistical significance in the SCI 3 and SCI 4 subgroups (Fig. 2b). Similarly, the BA level displayed a statistical significance in the SCI 4 subgroup (Fig. 2d). The levels of AA, PA, and BA did not exhibit significant differences among the various SCI subgroups (SCI 1–4). Moreover, the quantity of IBA was significantly elevated within the initial 3 months following the injury, reached its peak between 3 to 6 months post-SCI, and then gradually declined. However, even 6 to 12 months after injury, the IBA levels remained notably higher compared to the control group (Fig. 2c). Moreover, the IVA amount exhibited a pattern similar to that of IBA (Fig. 2e). In addition, VA and HA amounts showed no statistical difference in individual SCI subgroups when compared with the control group (Fig. 2f, g). Generally, the levels of certain SCFAs, particularly AA, PA, and BA, were significantly reduced following SCI, which seemed to be long-lasting.

### Fecal SCFA levers are altered in SCI patients with different injury segment

**Fig. 3:**
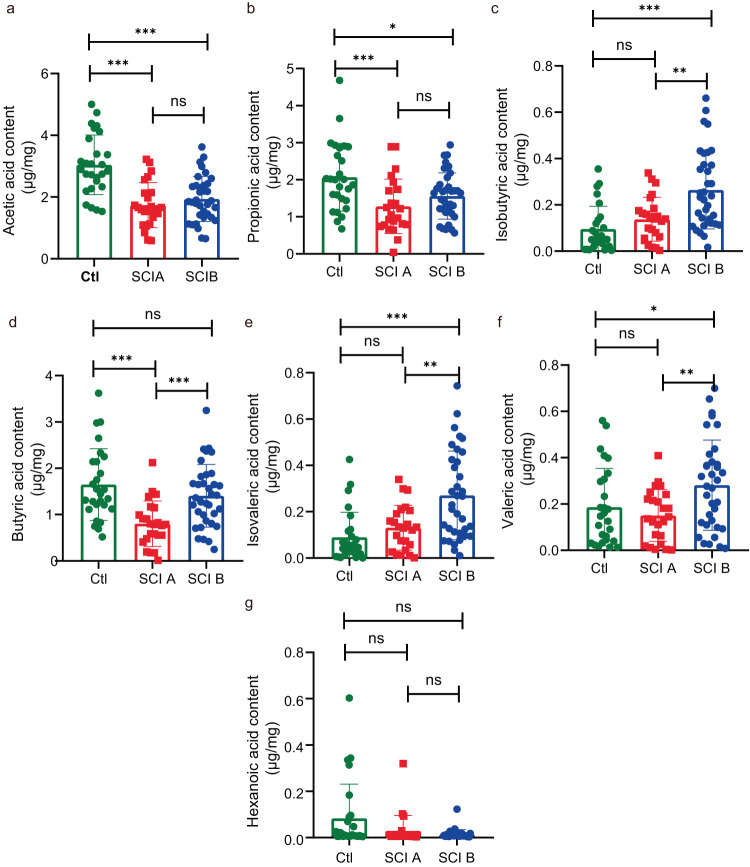
Aberrant SCFA levels in patients with SCI at different injury segment. Quantitative analyses of SCFA levels, including AA (**a**), PA (**b**), IBA (**c**), BA (**d**), IVA (**e**), IVA (**f**), HA (**g**), were performed by using GC-MS among different groups. One-way ANOVA followed by Tukey’s post-hoc test was used for comparisons among multiple groups and two-tailed paired *t* tests were used for comparisons between two groups. SEM were used to represent error bars. *p < 0.05; **p < 0.01, ns not significant (SCI A, injury above T6; SCI B, injury at or below T6).

SCI at any spinal cord segment affects intestinal autonomic control, and injury at a higher segment (above T6) results in almost complete loss of the spinal cord autonomic neural network that governs intestinal function. Our enrolled patients were classified into two groups based on lesion segment. The SCI A group comprised 24 patients (with injury segment above T6), while the SCI B group included 35 patients (with injury level at or below T6). In the SCI A and B groups, both AA and PA levels were notably diminished in comparison to the control group. Additionally, the BA level showed a decrease specifically in the SCI A group (Fig. 3a, b, d). In contrast, the IBA, IVA, and VA levels were significantly higher in the SCI B group than in the control group (Fig. 3c, e, f). Compared with the SCI A group, the SCI B group had increased IBA, BA, IVA, and VA levels (Fig. 3c–f). The HA level was not significantly different between the two groups (Fig. 3g). Our results showed that the lesion segment might have affected the levels of certain SFCAs.

### Correlation between SCFAs and microbiota

**Fig. 4:**
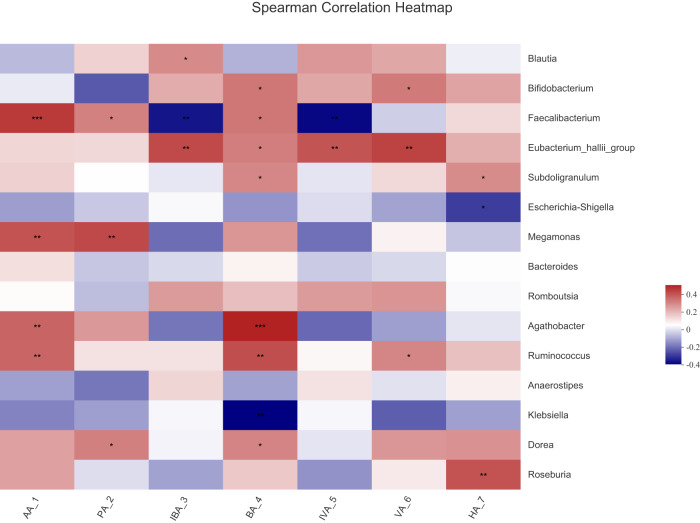
Correlations between SCFAs and community composition. A correlation heatmap analysis of SCFAs on the community composition of the control and SCI groups at the genus level.

Notably, the levels of certain SCFAs differed between the SCI and control groups, and SCFAs may represent potential interference targets in treating SCI. Consequently, correlation analysis was employed to investigate the association between SCFAs and the microbiota (Fig. 4). The correlation coefficient (R), R², and *p*-values resulting from the correlation analysis were presented in Table S1. The correlation heatmap analysis showed that the control group-enriched AA showed a positive relationship with *Faecalibacterium*, *Megamonas*, *Agathobacter*, and *Ruminococcus*. Similarly, PA showed a positive relationship with *Faecalibacterium*, *Megamonas*, and *Dorea*. In addition, a positive association was observed between BA and microflora, including *Bifidobacterium*, *Faecalibacterium*, *Eubacterium hallii*, *Subdoligranulum*, *Agathobacter*, *Ruminococcus*, and *Dorea*. In contrast, a negative association was observed between BA and *Klebsiella*. Moreover, a positive association was found between VA and microflora, including *Bifidobacterium*, *Eubacterium hallii*, and *Ruminococcus*. Consistently, *Subdoligranulum* and *Roseburia* positively interacted with HA, and *Escherichia* and *Shigella* negatively interacted with HA. IBA showed a positive relationship with *Blautia* and *Eubacterium hallii* and a negative relationship with *Faecalibacterium*. Similarly, IVA showed a positive relationship with *Eubacterium hallii* and a negative relationship with *Faecalibacterium*.

### SCFA treatment enhances locomotor recovery within SCI mice

**Fig. 5:**
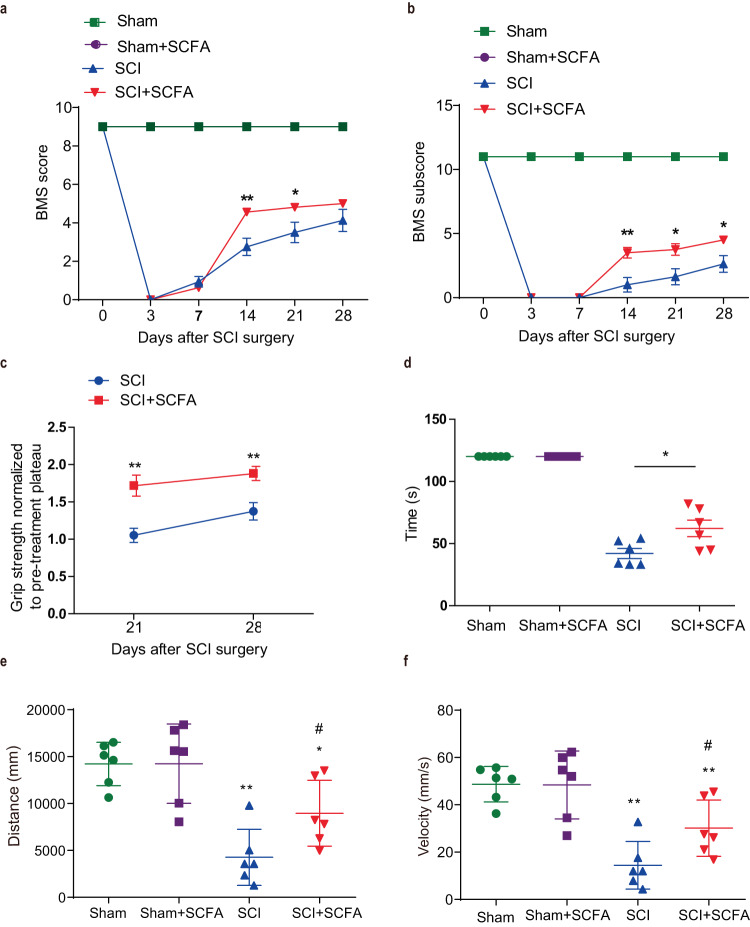
Effect of SCFA treatment on locomotor recovery. Time course of locomotor functional recovery as assessed by BMS (**a**) and BMS subscore (**b**). **c** Hindlimb grip strength. Data were normalized to pre-treatment (post-injury) baseline with 1 representing no difference after treatment, and values > 1 indicate improvement. **d** Latency to the time point when mice fell off a rotating rod (s) in the rotarod test. **e**, **f** Distance traveled or mean velocity during the open-field test. One-way ANOVA followed by Tukey’s post-hoc test was used for comparisons among multiple groups and two-tailed paired *t* tests were used for comparisons between two groups. SEM were used to represent error bars. *p < 0.05 compared with the sham group; **p < 0.01 compared with the sham group; ^#^*p* < 0.05 compared with the SCI group; ^##^*p* < 0.01 compared with the SCI group.

We hypothesized that SCFA supplementation might exert therapeutic effects on SCI mice. Thus, animals were administered a 4-week supplementation of drinking water containing acetate/butyrate/propionate mixture or control drinking water containing matched sodium chloride post-injury. The assessment of locomotor function recovery was conducted over a span of 4 weeks following the injury across the four groups. Notably, supplementation with SCFAs considerably facilitated the recovery of hindlimb locomotor function, with evident improvements observed from the 14th day post-injury when compared to the SCI group. This enhancement in the BMS scores continues until the 21st day post-injury (Fig. 5a). Moreover, a significant improvement in BMS subscores commenced from the 14th day post-injury and continued until the end of the experiment (Fig. 5b). The assessment of bilateral hindlimb motor function was conducted using a grip strength meter. The SCFA group had a significant improvement at 21 and 28 days post-injury when compared with the SCI group (Fig. 5c). Meanwhile, a rotarod test was performed to evaluate motor coordination and balance between forelimbs and hindlimbs. The pre-injury baseline test showed no significant difference between the two groups. Compared with the SCI group, the SCFA group showed a remarkably increased time spent on the rotarod at 4 weeks post-injury (Fig. 5d). An open field test was used to measure spontaneous locomotor activity at 28 days post-injury. The data showed that the total distance traveled and mean velocity between the SCI group and SCFA group were significantly different (Fig. 5e, f). Collectively, SCFA supplementation promoted motor coordination and balance in injured mice. In accordance with the behavioral analysis, histopathological examination through unbiased stereology at 4-week post-injury revealed that the SCI + SCFA group exhibited notably smaller lesion area, especially at 200 μm caudal to the lesion epicenter, in comparison to the SCI group, as evidenced by GFAP/DAPI staining (Fig. S6).

### SCFA treatment enhances neuronal survival and modulates synaptic plasticity

**Fig. 6:**
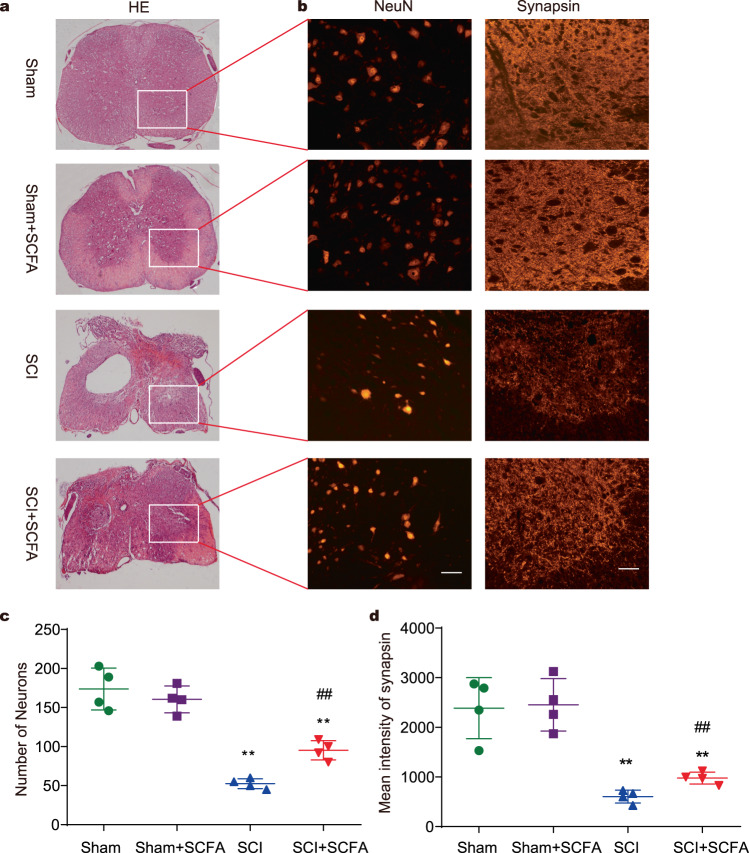
Effect of SCFA treatment on neuronal survival and synaptic formation following SCI. **a** HE staining and a squared inset of observed ventral horn. **b** Immunostaining of NeuN and synapsin in the T10 region of spinal cords in different groups. Quantification of NeuN-positive neuronal cell bodies (**c**), and synapsin immunoreactivity (**d**) of the ventral horn. Scale bar, 50 µm. One-way ANOVA followed by Tukey’s post-hoc test was used for comparisons among multiple groups and two-tailed paired *t* tests were used for comparisons between two groups. SEM were used to represent error bars. *p < 0.05 compared with the sham group; **p < 0.01 compared with the sham group; ^#^*p* < 0.05 compared with the SCI group; ^##^*p* < 0.01 compared with the SCI group.

To determine the morphological changes that might underlie behavioral functional recovery, immunofluorescent staining was performed on spinal cord sections to analyze how SCFAs affect neuronal survival and synaptic regeneration (Fig. 6). Mice treated with SCFAs displayed a higher count of NeuN-positive cells, as observed through the evaluation of NeuN-positive neurons in the ventral horn of SCI mice receiving either SCFAs or vehicle treatment (Fig. 6a, b). The quantification of neuronal cell bodies was conducted across the four groups (Fig. 6c). Synapsin-I (SYN) is related to synaptogenesis and the regulation of neurotransmitter release and plays a potential role in neurological regeneration and functional recovery^25,26^. Compared with the SCI group, the SCFA treatment group showed significantly increased SYN-positive signals within lesion areas at 4 weeks post-injury (Fig. 6b, d). These results suggest that SCFA treatment may enhance neuronal survival and promote synapse formation after injury.

### SCFA treatment exerts neuroprotective effect by reducing astrogliosis in the injured area of the spinal cord

**Fig. 7:**
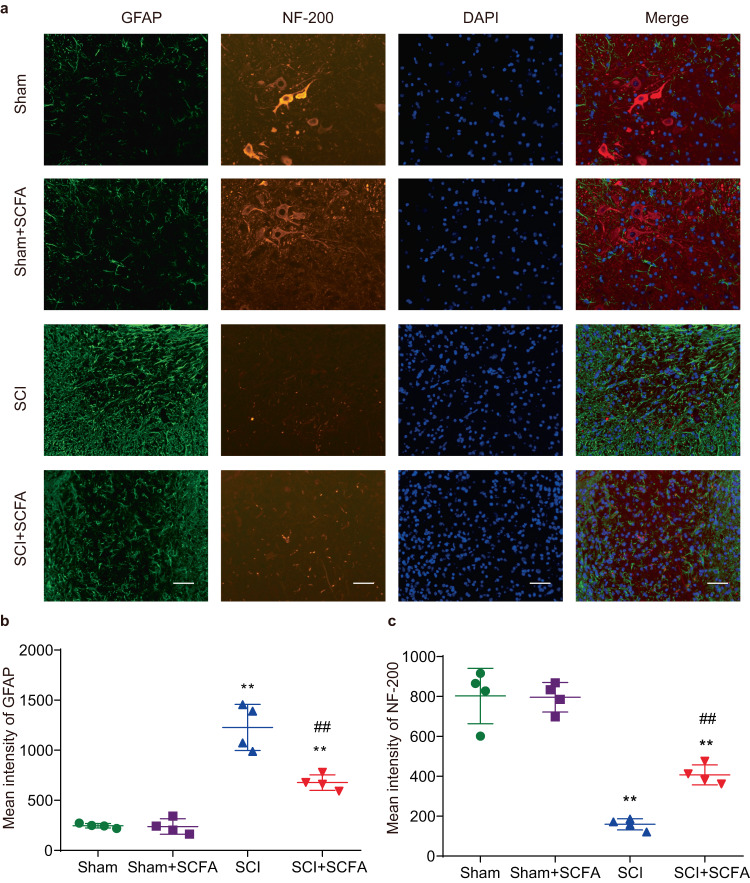
SCFA treatment exerts the neural protective function in the injured lesion area of the spinal cord. **a** Representative immunohistochemical staining images of NF (red) and GFAP (green) in different groups at day 28 post-injury. Semi-quantification of GFAP (**b**) and NF (**c**) intensities. One-way ANOVA followed by Tukey’s post hoc test was used for comparisons among multiple groups and two-tailed paired *t* tests were used for comparisons between two groups. SEM were used to represent error bars. *p < 0.05 compared with the sham group; **p <0.01 compared with sham group; #*p* < 0.05 compared with the SCI group; ##*p* < 0.01 compared with the SCI group.

Several studies demonstrated that astrogliosis occurs in an environment-dependent manner and impedes axonal regeneration^27,28^. Staining for GFAP, an astrocyte marker, demonstrated a notably elevated signal intensity in the lesion site of SCI mice. However, the administration of SCFAs reversed this elevation at the 28-day post-injury (Fig. 7a, b). Neurofilaments represent proteins specific to various cell types within the CNS, and have been utilized for the assessment of axonal and neural impairments^29,30^. NF staining showed that NF-positive signals within lesion areas were increased after SCFA supplementation 4 weeks post-injury when compared with the SCI group (Fig. 7a, c). These results suggest that SCFA treatment may inhibit astrogliosis and axonal impairments after traumatic SCI.

### SCFA treatment suppresses microglial activation and reduces neuroinflammation following SCI

**Fig. 8:**
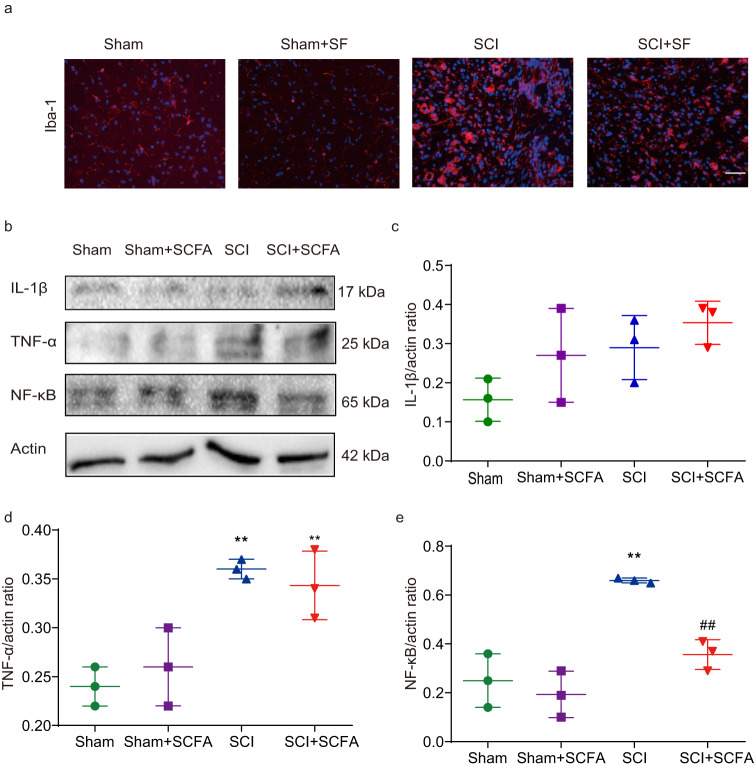
Effect of SCFA treatment on neuroinflammation following SCI. **a** Immunostaining of Iba-1 in the T10 region of spinal cords in different groups. **b** Expression of IL-1β, TNF-α, and NF-κB as analyzed using Western blot. The relative amounts of IL-1β (**c**), TNF-α (**d**), and NF-κB (**e**) were obtained using semi-quantitative analysis in different groups. One-way ANOVA followed by Tukey’s post-hoc test was used for comparisons among multiple groups and two-tailed paired *t* tests were used for comparisons between two groups. SEM were used to represent error bars. *p < 0.05 compared with the SCI group; **p < 0.01 compared with the sham group; ^##^*p* < 0.01 compared with the SCI group.

We performed Iba-1 staining to evaluate the effect of SCFA treatment on microglial activation post-injury. Following injury, Iba-1-positive microglial quantity was significantly upregulated. Compared with the SCI group, the SCFA group showed that Iba-1-positive microglial quantity significantly decreased within the traumatic lesion area 4 weeks post-injury (Fig. 8a). Western blotting was employed to validate these observations. Inflammatory factors, including IL-1β, TNF-α, and NF-kB (Fig. 8b), were determined. When compared to the control group, the SCI group exhibited notably heightened expression of TNF-α and NF-kB. However, the expression level of NF-κB was markedly reduced in the SCFA-treated group in comparison to the SCI group (Fig. 8c–e). Our findings indicate that supplementation with SCFAs attenuated neuroinflammation following SCI.

### SCFA treatment mediates neurotrophic factor expression

**Fig. 9:**
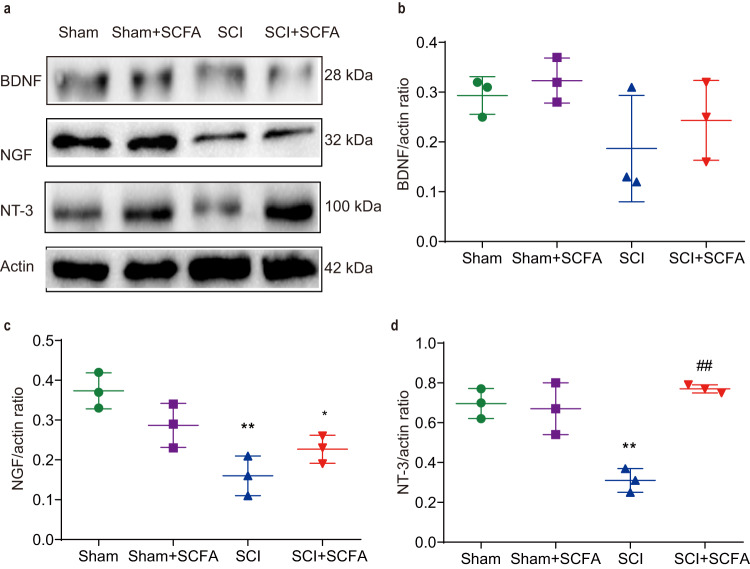
SCFA treatment alters the expression of neurotrophic factors in the spinal cords 4 weeks following injury. **a** Expression of BDNF, NT-3, and NGF as analyzed using Western blot. The relative amounts of BDNF (**b**), NGF (**c**), and NT-3 (**d**) were obtained using semi-quantitative analysis in different groups. One-way ANOVA followed by Tukey’s post hoc test was used for comparisons among multiple groups and two-tailed paired *t* tests were used for comparisons between two groups. SEM were used to represent error bars. *p < 0.05 compared with the sham group; **p <0.01 compared with the sham group; #*p* < 0.05 compared with the SCI group; ##*p* < 0.01 compared with the SCI group.

In addition to spinal cord pathophysiological alterations, we analyzed molecular changes through quantifying neurotrophic factor levels, such as BDNF, neurotrophin-3 (NT-3), and NGF within the injured spinal cord. As depicted in Fig. 9, the BDNF level was not significantly different among four groups; the levels of NT-3 and NGF exhibited a notable reduction in the SCI group in comparison to the control group. However, the supplementation of SCFAs notably increased the level of NT-3 when compared to the SCI group (Fig. 9d). Nonetheless, the NGF level was not significantly different between the SCI and SCFA groups (Fig. 9c). Our findings indicated that the protective effects of SCFA were possibly related to the increased levels of neurotrophic factors, particularly NT-3.

### SCFAs may exert neuroprotective effects by regulating intestinal microbiota or directly entering the circulating blood

Following a 4-week period of drinking water containing SCFAs, we analyzed the composition of gut microbiota in mice after SCI. The results indicated that, in comparison to the SCI group, SCI + SCFA mice exhibited discernible variations in their microbiota at both the phylum and genus levels (Fig. S7). We also conducted measurements of SCFAs in the serum. As illustrated in Fig. S8c, f, notable distinctions were observed in the concentrations of butyric acid and valeric acid between the sham group and the SCI group. Treatment with SCFAs led to a significant increase in the levels of butyric acid and valeric acid in SCI mice. Furthermore, SCFA treatment also considerably augmented the levels of propionic acid in SCI mice (Fig. S8b). In essence, supplementation with SCFAs notably modified the concentrations of serum SCFAs in SCI mice, distinct from the effects observed in sham mice. These changes in serum SCFAs are likely attributed partly to the direct supplementation of SCFA in the drinking water. On the other hand, the alterations in intestinal microbiota resulting from SCFAs supplementation may indirectly contribute to changes in serum SCFAs.

## Discussion

Emerging evidence suggests that gut dysbiosis is closely related to SCI development and severity^3,5^, whereas the detailed underlying mechanism between gut dysbiosis and SCI outcomes remains unknown. Thus, we performed clinical trials and animal experiments to explore this issue. GS-MS and 16 S rRNA sequencing were conducted for the collected fecal samples from patients with SCI, and the therapeutic effect of SCFA supplementation was examined in SCI mice. Our results showed that patients with SCI can be distinguished from healthy controls by using statistical variables of fecal SCFAs and bacterial populations. Importantly, a mouse model of SCI was used to evaluate the direct effect of SCFAs. SCFAs mediate neurological recovery from the molecular to behavioral levels. These data suggest that SCFAs, a part of the microbiome’s primary bioactive mediators, were significantly reduced after SCI, and SCFA supplementation offered a potential therapeutic option for post-SCI recovery.

Because of the increased appreciation of the gut–brain axis, gut–CNS communication through gut microbiota has attracted more attention. Many studies suggested that SCI altered gut microbial composition and abundance^3,5,6^. Although different inclusion criteria, including the injury degree, injury segment, injury course, and antibiotic use, coupled with eating habits and lifestyles, were applied in various clinical trials worldwide, relatively consistent conclusions have been drawn, that is, SCI leads to gut dysbiosis, particularly changes in bacterial populations that produce SCFAs. Nonetheless, no prior investigation has documented the levels of SCFAs in individuals with SCI. Our study has revealed a significant imbalance in gut microbiota accompanied by a deficiency of SCFAs in fecal samples from SCI patients. Furthermore, the SCFA data were classified based on the progression of injury and the spinal cord segment affected, and these two factors exhibited a discernible influence on SCFA levels. However, we did not observe alterations in both gut microbiota and SCFA levels within the same group of SCI patients across the transition from acute to chronic stages. As a result, forthcoming research endeavors should be meticulously designed with a longitudinal analysis and larger cohorts of patients. Moreover, correlation analyses showed that SCFAs, particularly AA, BA, and PA, exhibited a significant relationship with *Faecalibacterium, Agathobacter*, and *Megamonas. Faecalibacterium* is a major butyrate-producing bacterium^31,32^, affecting physiological function and homeostasis of the gastrointestinal tract. In our study, the abundance of *Faecalibacterium* was decreased in patients with SCI, which showed a positive relationship with AA, PA, and BA levels. *Agathobacter* is an anaerobic Gram-positive bacterium whose major fermentation products include acetate, butyrate, lactate, and hydrogen^33^. Our results showed that the abundance of *Agathobacter* was also reduced in patients with SCI, which was positively related with AA and BA levels. A previous study reported that *Megamonas* produced acetate and propionate based on glucose in vitro^34,35^. In our study, the abundance of *Megamonas* in patients with SCI was decreased, which showed a positive relationship with AA and PA levels. Among SCFAs, acetate, butyrate, and propionate were the main metabolites generated^17,36^. Although *Faecalibacterium* can directly produce butyrate and *Agathobacter* can directly produce butyrate and acetate, they may also affect SCFA levels through indirect interaction with other bacteria not producing SCFA. It is possible that there is a phylofunctional core of the SCFA-producing microbiota, and various bacteria may mutually influence each other.

Gut commensal bacteria generate SCFAs in the colon through anaerobic non-digestible carbohydrate fermentation, including resistant starch or dietary fibers^37^. SCFAs have a broad spectrum of beneficial properties that may improve neurological function in various CNS diseases via immune, vagal, endocrine, or additional humoral pathways^20,38–40^. Upon acute ischemic stroke, those with stroke severity had deficient SCFA-producing bacteria and decreased fecal SCFA levels^41^. Studies conducted on animals also demonstrated notably diminished plasma levels of SCFAs in mice subjected to fMCAO in comparison to mice subjected to sham operation. The supplementation of SCFAs exhibited improvement in post-stroke recovery through the modulation of immune cells present either within the brain or systemically^19^. Patients with multiple sclerosis (MS) showed a significantly reduced PA level, whereas PA treatment significantly enlarges gut-associated Treg cell population and subsequently reduces systemic immune reaction, leading to disease amelioration in an animal model of MS^42–44^. Furthermore, SCFA supplementation (PA) in patients with MS restored the Treg cell/Th17 imbalance by an immunomodulatory mechanism, resulting in disease improvement^45^. Our previous study showed intestinal microbial dysbiosis in patients with complete SCI^4^. The reprogramming of gut microbiota through fecal microbiota transplantation influenced the levels of SCFAs and ameliorated both locomotor and GI functions in mice with SCI. This beneficial effect is likely attributed to the anti-inflammatory properties of SCFAs^9^. Furthermore, the administration of sodium butyrate exerted neuroprotective effects on SCI mice via an anti-inflammatory mechanism^46^. These findings demonstrated that SCFAs could be potential therapeutic targets used to promote recovery after SCI.

Our study showed that SCFA supplementation promoted functional recovery in a contusion SCI mouse model. Moreover, SCFA supplementation prevents astrocytes and microglia activation while enhanced neurotrophic factor generation. Therefore, suppressing inflammation while upregulating trophic factors may collectively lead to a new balance of the spinal cord microenvironment contributing to repair and regeneration, finally prompting functional recovery. Furthermore, subsequent to a 4-week duration of drinking water enriched with SCFAs, there were noteworthy modifications observed in the composition of gut microbiota and the content of circulating SCFAs within mice afflicted with SCI. These alterations suggest that SCFAs treatment exerts a neuroprotective effect by replenishing serum SCFAs and indirectly influencing serum SCFAs levels through the remodeling of gut microbiota. Of notable significance, this study introduces an alternative therapeutic approach for individuals with SCI who may not be suitable candidates for fecal microbiota transplantation or exhibit limited responsiveness to probiotics supplementation. The execution of this investigation will additionally catalyze the exploration of dietary interventions and the development of pharmaceutical agents targeting gut microbiota metabolites. This, in turn, presents novel insights and empirical substantiation for the clinical translation of therapies addressing SCI.

In addition to the reduction of SCFA-producing bacteria, those discriminating taxa detected in this study did not fully match our previous results^4,47^. The discrepancy might be related to the different inclusion criteria of patients with SCI. Only male patients with chronic phase SCI were enrolled in the previous study. However, in the present study both male and female patients with SCI were enrolled, with a male/female ratio of approximately 5:1. Additionally, the enrolled patients in the current study included those in the chronic and acute phases. Thus, sex and different stages of the disease should be considered as variables in clinical trials of SCI, which may impact the characteristics of disease-related bacteria^48,49^. The choice of female mice in our animal experiments for SCI modeling was mostly considered from a practical standpoint^50^. Additionally, SCFAs were only examined for their neuroprotective effect in the SCI mouse model. Given the species differences, future clinical investigations should be performed to verify the effects of SCFAs.

Taken together, intestinal microbial composition is altered after SCI. SCFA-producing microbiota is significantly downregulated in patients with SCI compared with normal controls. Markedly reduced SCFAs were observed among SCI patients, which was associated with injury course and injury segment. Additionally, the supplementation SCFAs facilitated the recovery of functions, promoted the formation of axons, inhibited the activation of microglia, lowered the expression of NF-κB, and enhanced the secretion of NT-3. Our findings indicate that SCFAs derived from the microbiota can reinstate niche homeostasis in the aftermath of SCI through the attenuation of inflammation and the elevation of trophic factors. These effects may contribute to the facilitation of neurological repair, consequently leading to functional recovery.

## Methods

### Patients and control subjects

We enrolled 59 patients with SCI in the China Rehabilitation Research Center. We recruited 29 healthy participants from the nursing staff and patient’s relatives at the same hospital/center. All participants provided written informed consent to take part in the study. The Ethics Committee of China Rehabilitation Research Center approved our study protocols (Approval No.:2017–013–1).

Patient inclusion criteria were (1) SCI of neurological completeness [American Spinal Injury Association (ASIA) grade A], (2) patients aged 18–60 years, and (3) traumatic SCI. The exclusion criteria were (1) those who had stools of types 1, 2, 6, or 7 according to the Bristol Stool Form Scale; (2) using any pharmacological and Chinese traditional agents for improving evacuations; (3) used prebiotic, probiotic, antibiotic or bowel preparation treatment in the two-week period preceding the initiation of the study; (4) SCI of incompleteness, and (5) with metabolic diseases, gastrointestinal diseases, multiple sclerosis, and immune diseases. Before enrollment, all participants underwent general and full ASIA examinations.

Meanwhile, normal subjects were also enrolled based on the following criteria: (1) those aged 18–60 years and (2) without a history of metabolic diseases, immune disease, multiple sclerosis, or gastrointestinal diseases. Each participant was enrolled before sample collection, and informed consent was provided after being fully informed of the sampling procedure and research options.

### Sample collection and microbial DNA isolation

We obtained 86 fresh samples, including 27 and 59 from normal control subjects and patients with SCI. Subsequently, fresh stools were obtained into separate 2-mL sterile tubes. Then, the samples were immersed in liquid nitrogen, transferred into the freezer, and stored under −80 °C in the laboratory. The samples were collected within a 30-min period. Microbial DNA was isolated from collected stools using the E.Z.N.A.® Stool DNA Kit (Omega Bio-Tek, Norcross, GA, USA) following specific instructions.

### Polymerase chain reaction (PCR) amplification

PCR was performed to amplify bacterial 16 S rDNA gene’s V3–V4 region under the following conditions: 2 min at 95 °C; 30 s at 95 °C, 30 s at 55 °C, 30 s at 72 °C for 25 cycles, followed by 5-min extension at 72 °C with the primers 338 F 5′- ACTCCTACGGGAGGCAGCA-3′ and 806 R 5′- GGACTACHVGGGTWTCTAAT-3′. PCR reactions were performed in a 20-µL mixture containing 5× FastPfu Buffer (4 µL), 2.5 mM dNTPs (2 µL), respective primers (0.8 µL, 5 µM), FastPfu Polymerase (0.4 µL), and template DNA (10 ng) in triplicate.

### Illumina MiSeq sequencing

2% agarose gels were applied for extracting amplicons, followed by purification using the AxyPrep DNA Gel Extraction Kit (Axygen Biosciences, Union City, CA, USA) and quantification using QuantiFluor™-ST (Promega, USA). Afterward, amplicons in equimolar amounts were combined before paired-end sequencing (2 × 300 bp) using the Illumina MiSeq platform following specific instructions. Subsequently, we imported raw reads into the NCBI Sequence Read Archive database (Accession Number: PRJNA858945).

### Sequencing data processing

The quality of raw fastq files was screened through trimming and merging with FLASH based on the following criteria: (1) reads truncation at sites receiving a mean quality score of <20 within the 50-bp sliding window; (2) sequences with an overlap of >10 bp were merged based on corresponding overlaps with a mismatch of ≤2 bp; (3) separation of sequences for every sample following specific barcodes (exact matches) together with primers (two nucleotide mismatches were allowed), with the elimination of reads, including ambiguous bases.

### Detection of SCFAs

#### Preparation of standard solution for calibration

Stock solutions of each SCFA were initially prepared in MTBE at a concentration of 50 μg/mL. Internal standard (IS) solutions (2-methylpentanoic acid) were prepared in MTBE, at the concentration of 6 μg/mL. The external standard (SCFA) was employed for the purpose of dilution to create samples of gradient concentrations. The internal standard was then added to the samples of gradient concentrations and the establishment of the standard curve was done referring to a study by B Loye Eberhart et al. ^51^ In detail, the external standard working solutions underwent further dilution with pure MTBE, resulting in a series of standards with varying concentrations (0.005, 0.02, 0.025, 0.05, 0.1, 0.15, 0.5, 1, 2, 5, 10, 20 g/mL), each containing 0.3 μg/mL IS. The GC-MS system collected response data from the samples with gradient concentration standards. By utilizing the data pertaining to the ratio of responses and concentrations for both the internal and external standards, simulated standard curves were constructed. These standard curves were subsequently utilized for the quantitative assessment of the target substances within the sample.

### Biological sample preparation

All biological samples were stored at a temperature of –80 °C until they were ready for analysis. To analyze the serum samples, the serum was thawed in an ice bath and vortexed for 1 min. 100 μL serum was transferred into a 1.5 mL centrifuge tube and subsequently, the EP tube was introduced with phosphoric acid (100 μL, 0.5% v/v) under 3-min vortexing, followed by addition of MTBE solution containing IS (0.3 μg/ml), another 3-min vortexing, and a 5-min ultrasonic treatment. After 10-min centrifugation at 12,000 rpm at 4 °C, the supernatants were transferred into the vial prior to GC-MS analysis. After thawing in an ice bath, fecal sample (20 mg) was placed in the 2-mL centrifuge tube. Subsequently, the centrifuge tube was introduced with phosphoric acid (1 mL, 0.5% v/v) under 10-min vortexing, 5-min ultrasonic wave treatment and 10-min centrifugation at 12,000 rpm at 4 °C. Thereafter, supernatants (0.1 mL) were transferred into the 1.5-mL centrifuge tube, followed by addition of MTBE solution containing IS (0.3 μg/ml), followed by a 3-min vortexing, and a 5-min ultrasonic treatment. After 10-min centrifugation at 12,000 rpm at 4 °C, the supernatants were transferred into the vial prior to GC-MS analysis.

### Gas chromatography-mass spectrometry

The supernatants were subjected to analysis using gas chromatography-mass spectrometry (GC-MS/MS 7890B7000D; Agilent Technologies Inc.) with a silica capillary column (DB-FFAP, 30 m × 0.25 mm × 0.25 µm, Agilent J&W). The sample size injected was 2 µL (splitless), and the injector temperature was set at 200 °C. The initial oven temperature was set at 90 °C, and after holding for 1 min, it was increased to 100 °C at a rate of 25 °C/min. It was then further increased to 150 °C at a rate of 20 °C/min, and held for 0.6 min. Subsequently, the temperature was increased to 200 °C at a rate of 25 °C/min, and held for 0.5 min. Helium was used as the carrier gas at a flow rate of 1.2 mL/min. Mass spectrometry analysis was conducted under the following conditions: electron ionization (EI) source; quad temperature of 150 °C; ion source temperature of 230°C; transfer line temperature of 230 °C. Mass spectrometry detection was performed using both full-scan mode (EI at 70 eV, m/z values ranged from 40 to 300 Da, and acquisition scan time of 0.2 s) and multiple reaction monitoring (MRM) acquisition mode (collision gas rate of 1.5 mL/min, scan time of 62 ms for AA, 46 ms for PA, 46 ms for IBA, 43 ms for BA, 33 ms for IVA, 30 ms for VA, 62 ms for HA, and time window of 0.3 min). The quantification of fecal metabolites was carried out by using Metware Biotechnology Co., Ltd. (Wuhan, China).

### Animals

Adult female C57BL/6 N mice weighing 18–22 g were obtained from the Center of Experimental Animals of Capital Medical University (Beijing, China). The animals were raised in a standard environment, with a temperature of 22 ± 2 °C, the humidity of 55 ± 10%), and a light-dark cycle of 12:12 h, with free access to food and water. The Animal Care and Use Committee of Capital Medical University approved our animal protocols gained (Approval No.: AEEI-2022–111).

### SCI

We used 2% isoflurane for mouse anesthesia. Thereafter, laminectomy was performed to expose the T10 spinal cord, and 70-kilodyne contusion was performed using an Infinite Horizons Impactor (Precision Systems & Instrumentation, Lexington, KY, USA) before suturing of muscles and incision. Throughout the surgery and post-anesthesia recovery, the animals were put into the warming chamber until complete consciousness was reached. The mice received hydration through the subcutaneous administration of Ringer’s solution (0.5 ml) for a period of 5 days after the surgery. The urinary bladder was manually emptied two or more times each day during the entire duration of our study. Surgical interventions and postoperative animal care were conducted following guidelines and relevant policies for rodent survival surgery released by the Experimental Animal Committee of the Capital Medical University.

### Experimental groups

The animals were randomized into four groups (sham, sham + SCFA, SCI, SCI + SCFA). (1) The sham group underwent T10 laminectomy with no SCI and vehicle treatment; (2) the sham + SF group underwent T10 laminectomy with no SCI and 4-week SCFA treatment; (3) the SCI group had SCI and received vehicle treatment; and (4) the SCI + SF group had SCI and received a 4-week SCFA treatment. Each mouse was administered *ad libitum* with SCFA (consisting of 40 mM sodium butyrate, 25.9 mM sodium propionate, 67.5 mM sodium acetate) or vehicle control (containing 133.4 mM sodium chloride) dissolved into drinking water for 4 weeks based on the previous description^19,42^.

The following were the numbers of mice allocated into each group and the anesthesia method adopted for various experiments. Immunohistochemical analysis on spinal cords, *n* = 4 in each group; mice were anesthetized with pentobarbital sodium (40 mg/kg) and then perfused with paraformaldehyde to extract spinal cord tissue for subsequent sectioning. GFAP staining for lesion size analysis, *n* = 4 in each group; mice were anesthetized with pentobarbital sodium (40 mg/kg) and then perfused with paraformaldehyde to extract spinal cord tissue for sectioning. Western blotting analysis of spinal cords, *n* = 3 in each group; mice were anesthetized with pentobarbital sodium (40 mg/kg) and then perfused with saline to extract spinal cord tissue for total protein extraction. The same batch of mice (*n* = 6 in each group) were also used for behavioral evaluation. The experiment endpoint was 4 weeks following SCI.

### Behavioral test

This study adopted the Basso Mouse Scale (BMS) for scoring hindlimb movements based on the prior description^52^. The animal assessment was completed within the open field for 4 min preoperatively, and on days 3, 7, 14, 21, and 28 postoperatively. Bilateral hindlimb performances were rated, and the mean was calculated to generate the BMS scores and subscores. The subscores covered stepping frequency, coordination, paw position, trunk stability, and tail position analyses, which represent a part of the BMS scoring system.

The open-field assay has been extensively applied in assessing locomotor activity. It was performed in this study based on previous descriptions after slight modifications^53^. In summary, the mice engaged with the open arena (50 × 50 × 50 cm, L × W × H) for a duration of 5 min. Following this, the assessment was conducted under subdued illumination (20 lx). Prior to the subsequent trial, the open field test box was cleansed using 70% ethanol. A mounted camera was employed to record each experiment. The evaluation encompassed measurements of the total distance covered and the average speed, which were analyzed using the TopScan software (Clever Systems, Reston, VA).

This study also used the Rotarod test to assess rodent motor coordination based on previous descriptions after slight modifications^54^. In brief, each mouse was put onto the 30-mm rotating rod within the five-lane Rotarod device, accelerating at 0–30 rpm in 90 s, and one test lasted for 120 s at most, with five trials per session. The trials were terminated if the mice fell off or clung to the rod. The latency of the mice on the rotating rod was recorded and calculated based on the time they fell off during each trial; then, the mean was calculated to obtain the final score for every session. Each animal received 3-day training (five times/day) before SCI onset.

Grip strength was evaluated using a grip force meter (Columbus Instruments, Columbus, OH, USA). In each trial, the animals were placed onto the apparatus and then pulled horizontally until their grip was released. Each mouse underwent five trials with intervals of 10 s, and the average values were calculated for various time points after excluding the highest and lowest measurements.

### Immunohistochemistry and quantitative image analysis

Each mouse was administered with 40 mg/kg pentobarbital sodium i.p. for anesthesia 4 weeks post-SCI, followed by perfusion using 0.1 M PBS (pH 7.4, 37 °C) and 4% (w/v) paraformaldehyde fixation within the 0.1 M PBS. Subsequently, 20-µm frozen spinal cord tissue sections were sliced using the cryostat microtome (Leica CM 3500; Wetzlar, Germany) before mounting them onto gelatin-coated glass slides. After 10-min equilibration within the 0.1 M Tris-buffered saline, the sections were blocked using 10% normal goat serum in PBS for 1 h. In contrast, spinal cord sections were incubated for 1 h after adding primary antibodies, including mouse monoclonal anti-NeuN (1:100, ab177487, Abcam), rabbit polyclonal anti-NF (1:200, 2836 S, Cell Signaling), rabbit polyclonal anti-synapsin (1:100, ab64581, Abcam), rabbit polyclonal anti-Iba-1 (1:100, GeneTex, GTX100042), and rabbit polyclonal anti-GFAP (1:500, Sigma, HPA056030). Afterward, the sections were washed with PBS, incubated using secondary antibodies, and cover-slipped using the glycerin-mounting medium before examination under the fluorescence microscope. Images were captured using identical exposure settings for each experimental group. The images were taken at the ventral horn area from consistent positions. For imaging, five sections per mouse were chosen around the site of the spinal cord injury, maintaining an interval of 100 μm between sections. Quantitative evaluation of neuron counts and the average intensity of synapsin, GFAP, and NF was performed using Image Pro Plus 7.0 software (Media Cybernetics, Silver Spring, MD, USA). The statistical analysis was conducted using the average values from all five sections of each animal. Analysis of the lesion size was carried out by using Image J software.GFAP stained tissue sections located at 0 μm, 200 μm, 400 μm, and 1 mm rostral and caudal to the lesion epicenter, respectively, were analyzed, and the area of the lesion was delineated using the polygon selection tool in Image J. Four animals were analyzed in each experimental group.

### Western-blotting assay

The 1-cm long spinal cord (at T9–T11, which extended 5 mm to caudal and cranial ends, separately) was collected. To separate total proteins, a lysis buffer (Beyotime, China) was added to lyse tissue homogenates for 1 h, followed by 8-min centrifugation at 14,000 × *g* at 4 °C. Afterward, protein concentrations within supernatants were analyzed using the protein assay kit (Pierce, Rockford, IN, USA). Following this, protein samples (50 µg) were loaded onto a 12% sodium dodecyl sulfate-polyacrylamide gel electrophoresis setup to facilitate separation. Subsequently, the proteins were transferred onto polyvinylidene difluoride membranes. Following a 1-h blocking step using a 5% defatted milk solution in Tris-buffered saline containing 0.05% Tween-20 (TBST), antibodies against brain-derived neurotrophic factor (BDNF) (1:500, Abcam, ab108319), neuronal growth factor (NGF) (1:500, Abcam, ab52918), neurotrophin-3 (NT-3) (1:500, Abcam, ab16640), interleukin-1β (IL-1β) (1:100, Abcam, ab9722), tumor necrosis factor-α (TNF-α) (1:100, Abcam, ab6671), or nuclear factor kappa B (NF-κB) (1:100, Abcam, ab16502) were added for an overnight incubation at 4 °C. After rinsing thrice with TBST, the membranes were further incubated using specific horseradish peroxidase-labeled secondary antibodies, with β-actin (1:1000, Abcam, ab8227) as the endogenous reference. Bands were visualized using enhanced chemiluminescence, whereas imaging was acquired using ChemiDoc MP System (Bio-Rad, Hercules, CA, USA). Relative band intensities were quantified using Image Lab4.1 (Bio-Rad, Hercules, CA, USA). All blots or gels derive from the same experiment and that they were processed in parallel. Original blots are provided in Supplementary Information.

### Bioinformatics and statistical analyses

Sequence analysis was conducted using QIIME2^55^, with additional analyses conducted in R version 3.3.1. Multiplexed single-end sequencing reads (a total of 4,749,608) were imported into QIIME2. The DADA2^56^ plugin within QIIME2 was utilized to ‘denoise’ the sequencing reads by removing sequences with expected errors greater than 2 and truncating sequences at the first base with a base mass value less than or equal to 0. This step filtered out noise, corrected errors in marginal sequences, eliminated chimeric sequences and singletons, and finally dereplicated the resulting sequences. This process yielded high-resolution amplicon sequence variants (ASVs) for subsequent analysis. Taxonomy was assigned to sequences using a naive Bayes classifier and the q2-feature classifier plugin, utilizing a reference database (SILVA Release 138). A total of 1,665,320 high-quality reads were generated, ranging from 39,863 to 14,056 per sample, with an average of 20,309 reads per sample. After rarefying the sample reads to 11,419, a total of 936,358 ASVs from 82 samples were included for downstream analysis of the microbial community.

Alpha and beta diversity analyses were performed using the diversity plugin in QIIME2. The Chao index (R-3.3.1(stat)), Shannon’s index (R-3.3.1(stat)), and rarefaction curves (R-3.3.1(vegan)) were employed to characterize alpha-diversity at the ASV level. Beta-diversity analysis was conducted through principle coordinate analysis (PCoA) (R-3.3.1(vegan)) using a weighted UniFrac dissimilarity matrix. Methods, including the Wilcoxon rank-sum test and LEfSe were employed to identify bacterial taxa with differing abundances between groups (R-3.3.1 (stat)). Spearman correlation analysis was conducted to assess the correlation between important bacterial taxa and SCFAs. A heatmap (R-3.3.1 (stat)) was constructed to explore the potential relationship between gut microbiota and metabolites. The functional microbial profiles were predicted and analyzed based on PICRUSt2 (v2.2.0-b). Data analysis was carried out on the free online Majorbio Cloud Platform (http://www.majorbio.com).

Data were expressed as means and standard error of the mean and examined using SPSS 17.0 (SPSS Inc., Chicago, IL, USA). All data analysis was conducted blindly with respect to experimental conditions. Individual samples/animals were randomized through numbering. We performed all statistical tests using GraphPad Prism 9.0 Software. While we assumed a normal data distribution, formal testing was not conducted. Statistical analysis was performed using one-way ANOVA followed by Tukey’s multiple comparisons test and two-tailed paired *t* tests were used for comparisons between two groups. A significance level of *p* < 0.05 was considered statistically significant.

### Reporting summary

Further information on research design is available in the Nature Research Reporting Summary linked to this article.

### Supplementary information

Supplementary material

Reporting Summary

## Data Availability

DNA sequencing data produced as part of the present work were imported into the NCBI Sequence Read Archive database (Accession Number: PRJNA858945).
