# Supplementary material for: Spinal cord injury-induced gut dysbiosis influences neurological recovery partly through short-chain fatty acids
Source: NPJ Biofilms Microbiomes. 2023 Dec 14;9:99. doi: 10.1038/s41522-023-00466-5 (PMC10719379; doi:10.1038/s41522-023-00466-5)

Supplementary materials for

Spinal cord injury-induced gut dysbiosis influences neurological recovery partly through short-chain fatty acids

Yingli Jing[a,b], Degang Yang[a,b,c*], Fan Bai[a,b], Qiuying Wang[a,b], Chao Zhang[d], Yitong Yan[a,b], Zihan Li[a,b], Yan Li[a,b], Zhiguo Chen[b,e&], Jianjun Li[a,b&], Yan Yu[a,b&]*

[a] *China Rehabilitation Science Institute, China Rehabilitation Research Center, Beijing Key Laboratory of Neural Injury and Rehabilitation, and School of Rehabilitation Medicine, Capital Medical University, Beijing, 100068, China*

[b] *Center of Neural Injury and Repair, Beijing Institute for Brain Disorders, Beijing, 100068, China*

[c] *Department of Spinal and Neural Function Reconstruction, Beijing Bo'ai Hospital, Beijing, 100068, China*

[d] *Department of Neurosurgery, Linyi People's Hospital, Shangdong, 276034, China*

[e] *Cell Therapy Center, Beijing Institute of Geriatrics, Xuanwu Hospital Capital Medical University, National Clinical Research Center for Geriatric Diseases, and Key Laboratory of Neurodegenerative Diseases, Ministry of Education, Beijing, 100053, China*

* Yingli Jing and Degang Yang contributed equally to this work.

&Corresponding authors: Zhiguo Chen (chenzhiguo@gmail.com, Tel: 8610-15810702747),

Jianjun Li (crrcljj@163.com, Tel: 8610-13910500026)

and Yan Yu (yuyancrrc@163.com, Tel: 8610-13426262203)

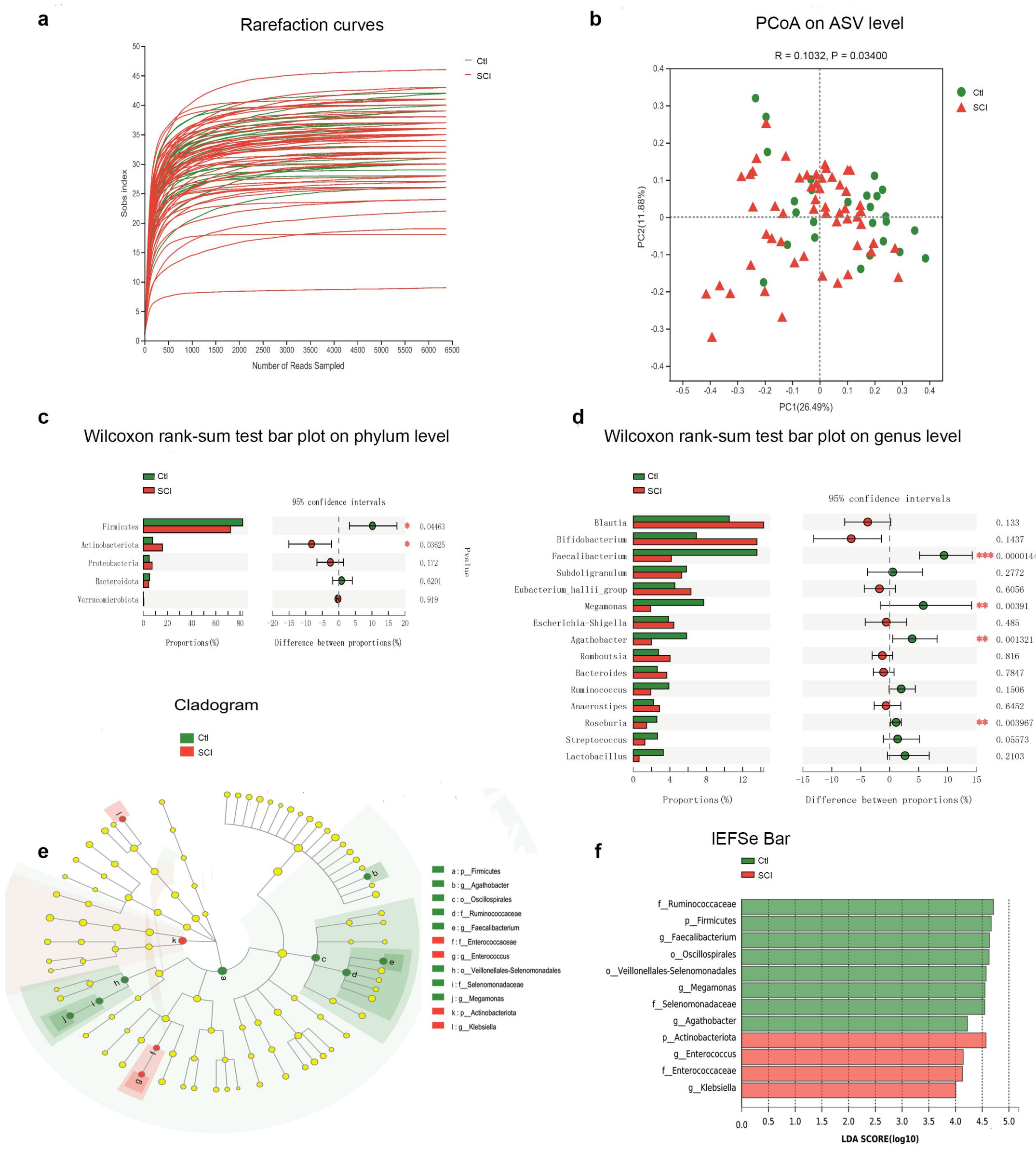

Supplementary Figure 1. Gut microbiome composition profiles differ between the control and SCI groups. **a** Rarefaction curves for ASV levels in the four groups. **b** Scatter plots of principal coordinate analysis (PCoA) scores showing similarity of the bacterial communities based on the weighted Unifrac distance. **c** Quantitative analyses of the relative abundances of bacteria at the phylum level. **d** Quantitative analyses of the relative abundances of bacteria at the genus level. Statistical significance was evaluated using Wilcoxon rank-sum test. SEM were used to represent error bars. *p < 0.05 compared with the SCI group; **p < 0.01 compared with the SCI group;

***p < 0.001 compared with the SCI group. **e** The LEfSe method was used to build a cladogram to analyze the phylogenetic distribution of fecal microbiota related to patients with SCI and healthy subjects. **f** LEfSe analysis showed significant differences in bacterial abundances in the fecal microbiota between the SCI and control groups. Linear discriminant analysis scores (log10) > 4 and p < 0.05 are listed.

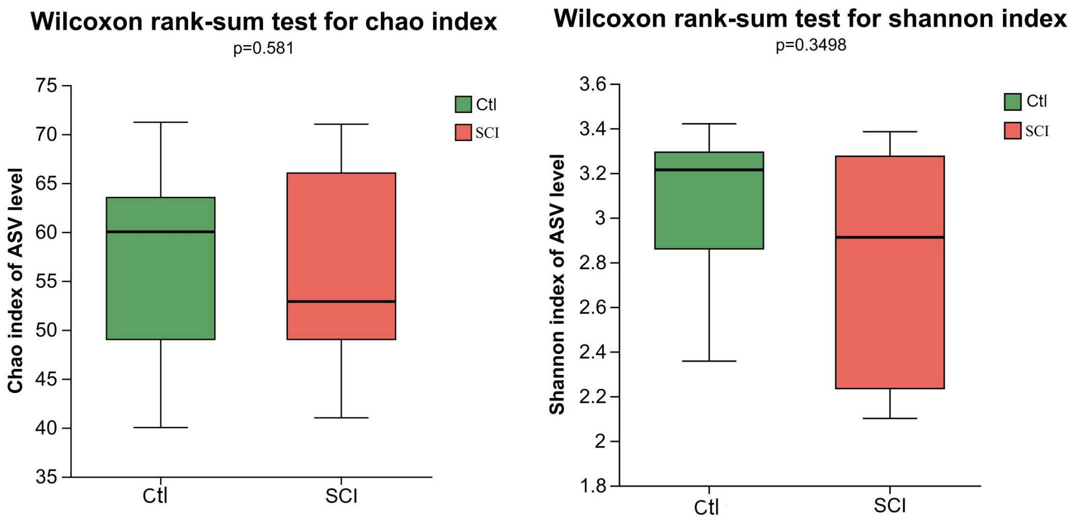

Supplementary Figure 2. The α- diversity (chao index and shannon index) between the control and SCI groups. Comparison of the richness (assessed by chao index) and the diversity (assessed by shannon index) based on the ASV levels in the two groups. Statistical significance was evaluated using Wilcoxon rank-sum test. SEM were used to represent error bars.

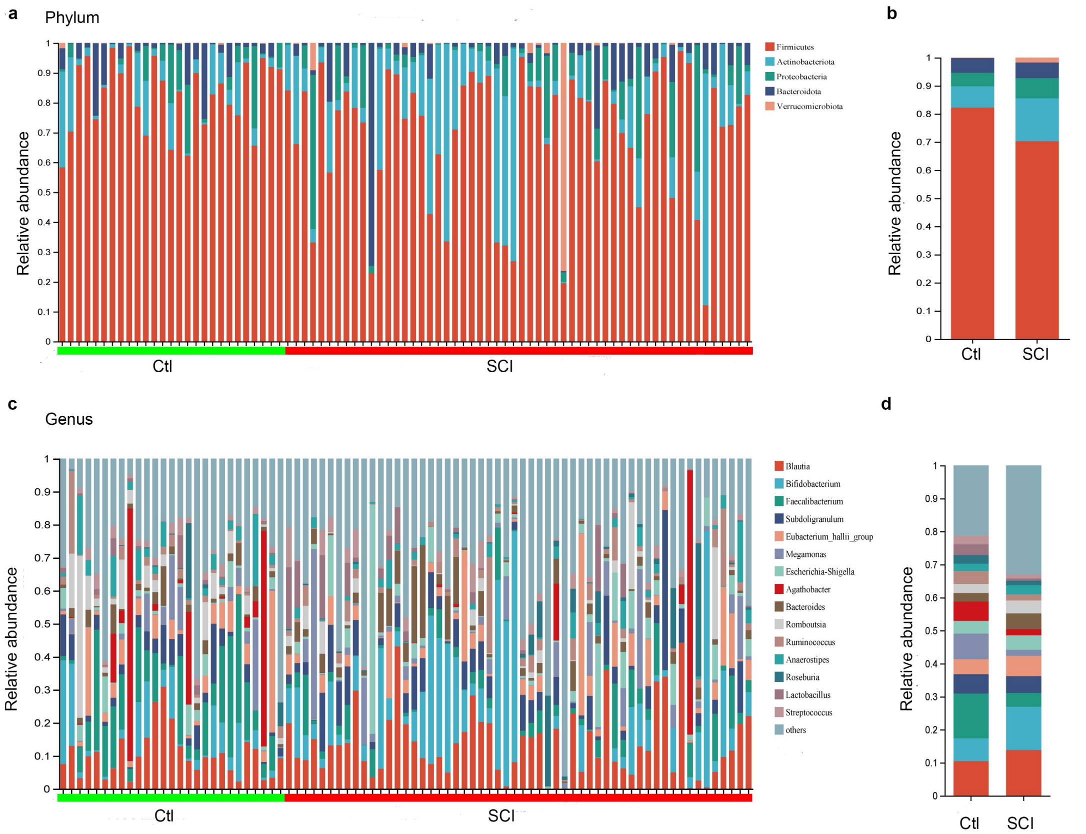

Supplementary Figure 3. Compositional structure of the gut microbiota between the control and SCI group. **a** Bacterial composition of the different communities at the phylum level. **b** The average relative abundance of gut microbiota at the phylum level in the two groups. **c** Bacterial composition of the different communities at the genus level. d The average relative abundance of gut microbiota at the genus level in the two groups.

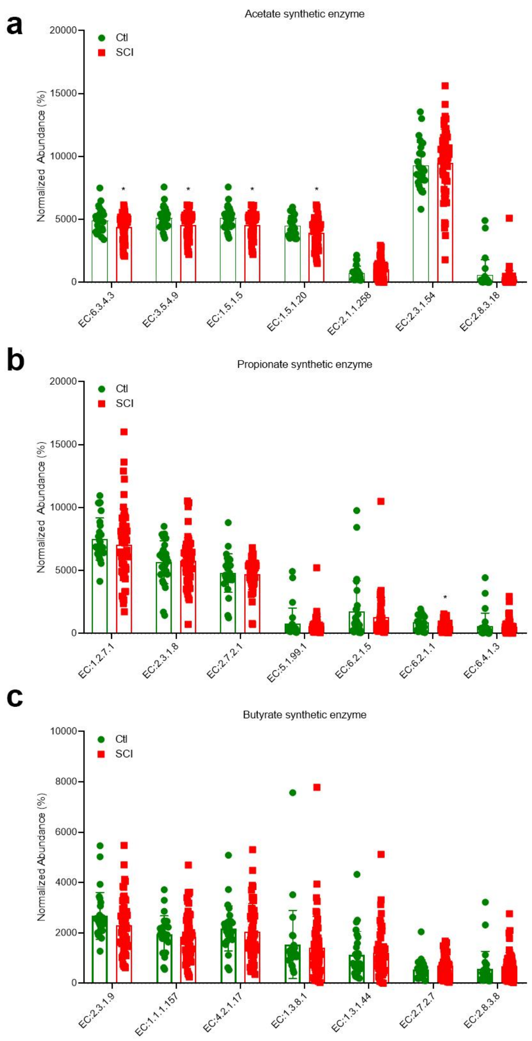

Supplementary Figure 4. The abundances of SCFA-related enzymes differ between the control and SCI groups. **a-c** Relative abundance of acetate, propionate, and butyrate synthesis-related enzymes from analysis results using PICRUSt2. The expression of some metabolic enzymes, such as EC:6.3.4.3, EC:3.5.4.9, EC:1.5.1.5, EC:1.5.1.20 (acetate) and EC6.2.1.1 (propionate), were obviously higher in Control group. However, most of butyrate synthesis-related enzymes were no significant difference between the two groups.Two-tailed paired t tests were used for comparisons between two groups. SEM were used to represent error bars. *$p < 0.05$.

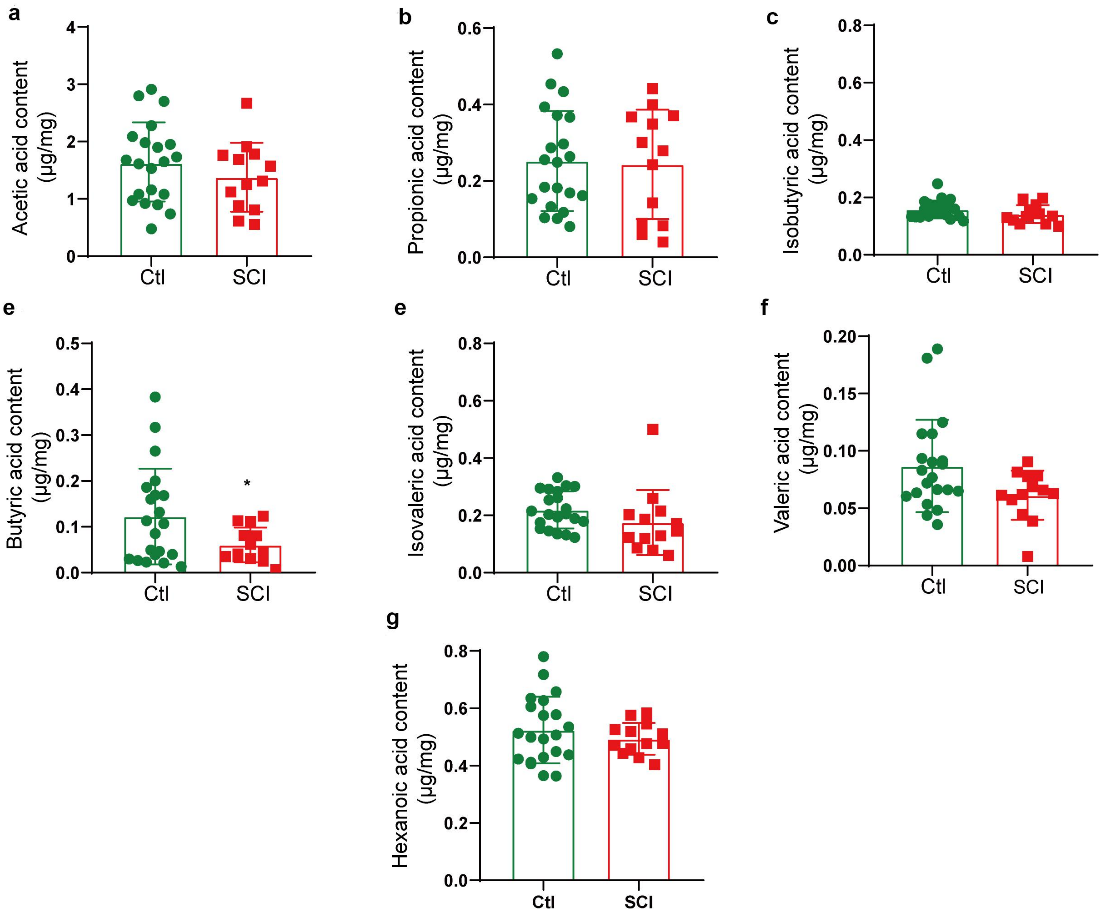

Supplementary Figure 5. Altered levels of circulating SCFAs in patients with SCI. Quantitative analyses of SCFA levels, including acetic acid (AA) (**a**), propionic acid (PA) (**b**), isobutyric acid (IBA) (**c**), butyric acid (BA) (**d**), isovaleric acid (IVA) (**e**), valeric acid (VA) (**f**), and hexanoic acid (HA) (**g**) were performed by using a gas chromatography–mass spectrometry (GC-MS) between the control and SCI groups. Two-tailed paired t tests were used for comparisons between two groups. SEM were used to represent error bars. *p < 0.05. (Con: n = 21; SCI: n = 13)

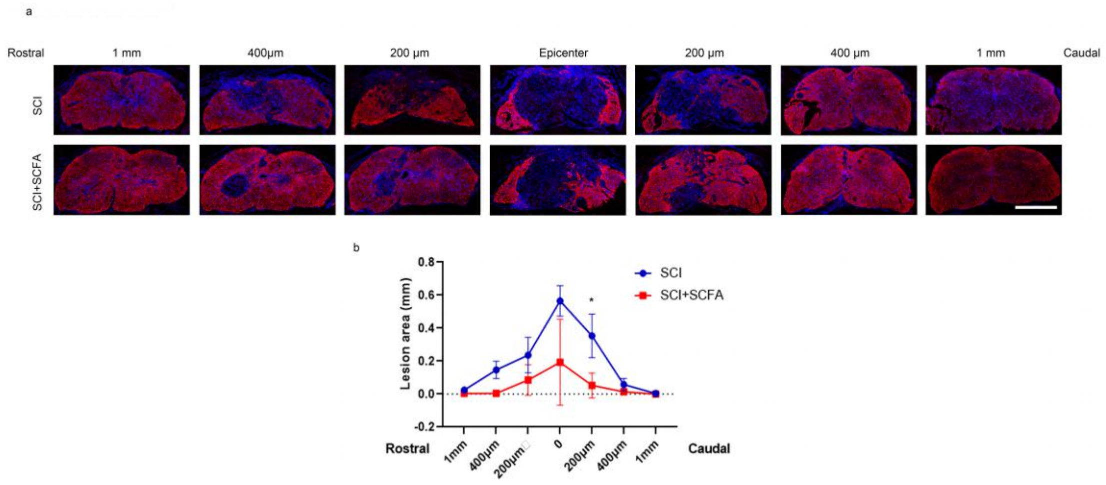

Supplementary Figure 6. Lesion size is measured and compared between the two groups. **a** Representative images located at 0 µm, 200 µm, 400 µm, and 1 mm rostral and caudal to the lesion epicenter, respectively, from SCI and SCI+SCFA groups are shown. **b** Lesion areas are quantified by using Image J. Two-tailed paired t tests were used for comparisons between two groups. SEM were used to represent error bars.*$p < 0.05$. Scale bar = 500 µm.

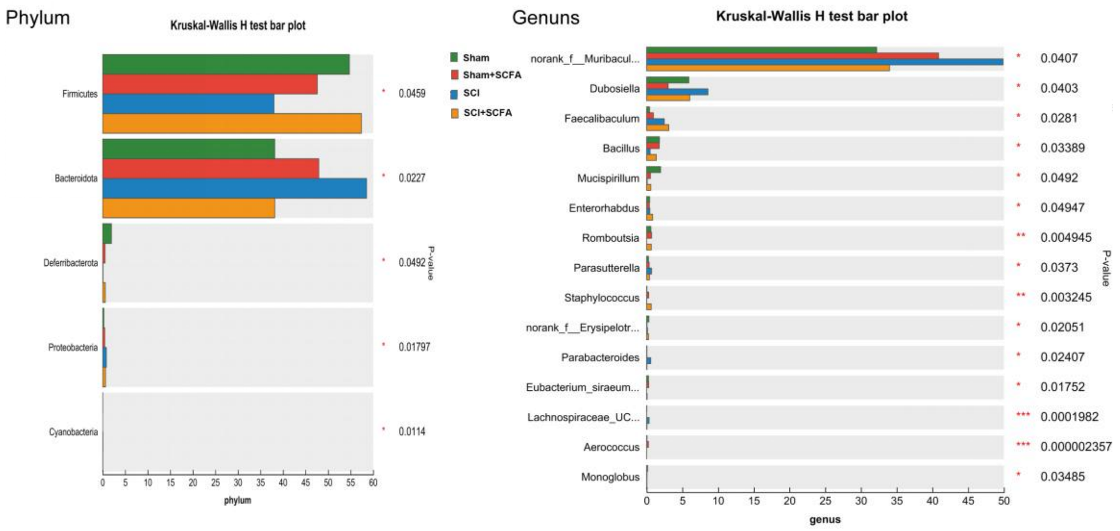

Supplementary Figure 7. Treatment with SCFAs alters the gut bacterial composition following SCI. Quantitative analyses of the relative abundances of bacterial composition of the different communities at the phylum and genus levels. Statistical significance was evaluated using Kruskal-Wallis H test.

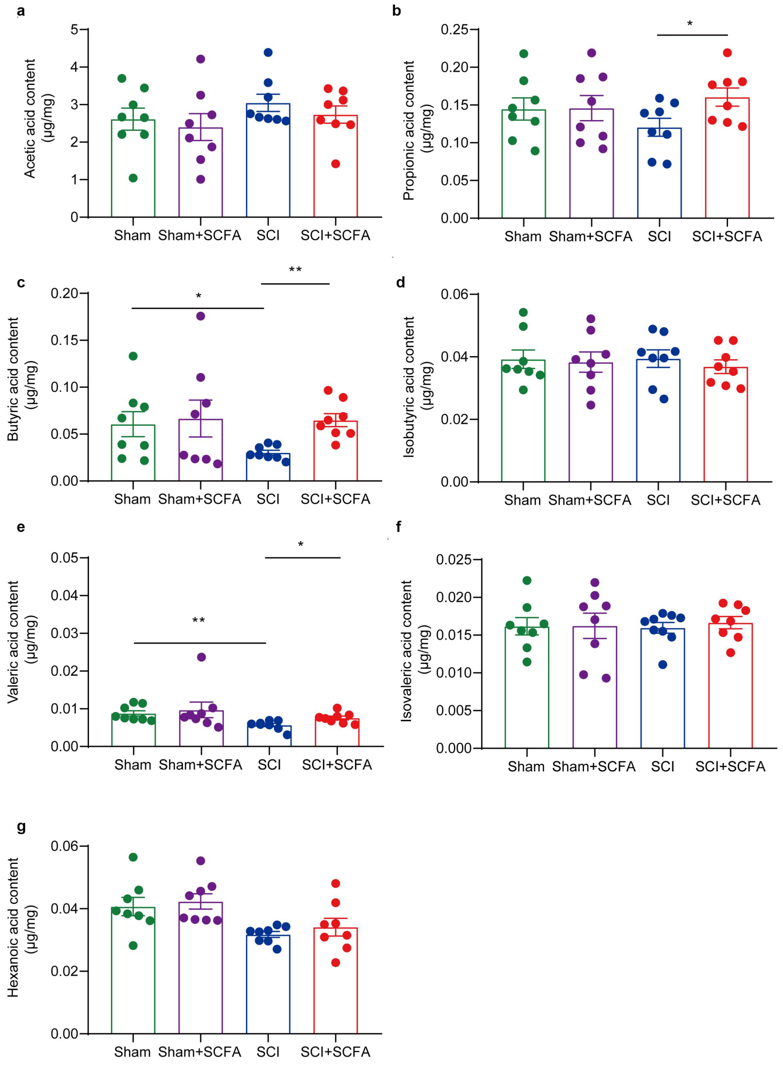

Supplementary Figure 8. Treatment with SCFAs in drinking water alters the content of serum SCFAs. Quantitative analyses of SCFA levels, including acetic acid (AA) (**a**), propionic acid (PA) (**b**), isobutyric acid (IBA) (**c**), butyric acid (BA) (**d**), isovaleric acid (IVA) (**e**), valeric acid (VA) (**f**), and hexanoic acid (HA) (**g**) were performed by using a gas chromatography–mass spectrometry (GC-MS) between the control and SCI groups. One-way ANOVA followed by Tukey's post-hoc test was used for comparisons among multiple groups and two-tailed paired t tests were used for comparisons between two groups. SEM were used to represent error bars. *$p < 0.05$. **$p < 0.01$.

Supplementary Table1. The correlation coefficient (R), R^2 and p-value of correlation analysis.

R

	AA_1	PA_2	IBA_3	BA_4	IVA_5	VA_6	HA_7
g__Blautia	-0.040439288	0.19874307	0.292431471	-0.057895896	0.265708366	0.232627205	0.010851829
g__Bifidobacterium	-0.070465972	-0.269877945	0.157076704	0.158955365	0.150905895	0.156691769	0.083735277
g__Faecalibacterium	0.361663335	0.246087896	-0.281397073	0.221785398	-0.287287858	-0.010613901	0.152364882
g__Eubacterium_hallii_group	0.037244488	0.075113629	0.406151097	0.208279493	0.399507392	0.374241472	0.23164573
g__Subdoligranulum	0.103899356	0.078270541	0.038292658	0.229500404	0.029699378	0.104765762	0.203345604
g__Escherichia-Shigella	-0.10702323	-0.068246529	0.040194991	-0.102353807	0.006540858	-0.066042325	-0.164069266
g__Megamonas	0.315508401	0.340869821	-0.179401957	0.171766614	-0.174460471	0.024594432	-0.058352876
g__Romboutsia	0.096025481	-0.00970797	0.192740334	0.202101829	0.197095298	0.201570964	0.031651288

g__Bacteroides	0.036833962	-0.124138126	-0.052399182	0.004014726	-0.05506307	-0.021433855	0.072123094
g__Agathobacter	0.288233262	0.225702167	-0.116753548	0.396789148	-0.119520079	-0.081193127	0.054681174
g__Anaerostipes	-0.099106431	-0.121139891	0.119727545	-0.089851344	0.093258636	-0.024433363	0.006122565
g__Ruminococcus	0.192766253	0.029460002	0.044816168	0.250460408	0.020275706	0.197361952	0.112551746
g__Klebsiella	-0.092277527	-0.058330951	-0.007940527	-0.235999982	-0.001985087	-0.18864093	-0.138900275
g__norank_f__Eubacterium_coprostanoligenes_group	-0.030045293	-0.009593437	0.321776219	0.256224312	0.346512257	0.485240793	0.228538835
g__Dorea	0.155526711	0.251153477	0.062489352	0.195243811	0.042781256	0.195033492	0.258164043

R2

	AA_1	PA_2	IBA_3	BA_4	IVA_5	VA_6	HA_7
g__Blautia	0.001635336	0.039498808	0.085516165	0.003351935	0.070600936	0.054115416	0.000117762
g__Bifidobacterium	0.004965453	0.072834105	0.024673091	0.025266808	0.022772589	0.02455231	0.007011597
g__Faecalibacterium	0.130800368	0.060559253	0.079184313	0.049188763	0.082534313	0.000112655	0.023215057
g__Eubacterium_hallii_group	0.001387152	0.005642057	0.164958714	0.043380347	0.159606156	0.140056679	0.053659744
g__Subdoligranulum	0.010795076	0.006126278	0.001466328	0.052670435	0.000882053	0.010975865	0.041349435
g__Escherichia-Shigella	0.011453972	0.004657589	0.001615637	0.010476302	4.28E-05	0.004361589	0.026918724
g__Megamonas	0.099545551	0.116192235	0.032185062	0.02950377	0.030436456	0.000604886	0.003405058
g__Romboutsia	0.009220893	9.42E-05	0.037148836	0.040845149	0.038846556	0.040630853	0.001001804
g__Bacteroides	0.001356741	0.015410274	0.002745674	1.61E-05	0.003031942	0.00045941	0.005201741
g__Agathobacter	0.08307	0.05094	0.01363	0.15744	0.01428	0.00659	0.00299

	8414	1468	1391	1628	5049	2324	0031
g__Anaerostipes	0.009822085	0.014674873	0.014334685	0.008073264	0.008697173	0.000596989	3.75E-05
g__Ruminococcus	0.037158828	0.000867892	0.002008489	0.062730416	0.000411104	0.03895174	0.012667896
g__Klebsiella	0.008515142	0.0034025	6.31E-05	0.055695992	3.94E-06	0.0355854	0.019293286
g__norank_f__Eubacterium_coprostanoligenes_group	0.00090272	9.20E-05	0.103539935	0.065650898	0.120070744	0.235458627	0.052229999
g__Dorea	0.024188558	0.063078069	0.003904919	0.038120146	0.001830236	0.038038063	0.066648673

p-value

	AA_1	PA_2	IBA_3	BA_4	IVA_5	VA_6	HA_7
g__Blautia	0.720011515	0.075292994	0.008069337	0.607674874	0.016508569	0.036629767	0.923400441
g__Bifidobacterium	0.531895991	0.01482837	0.16138528	0.156368322	0.178703153	0.162427804	0.457354067
g__Faecalibacterium	0.000908486	0.026791479	0.010932216	0.046601877	0.009309617	0.92507499	0.174491151
g__Eubacterium_hallii_group	0.741320629	0.505118533	0.000168514	0.062055436	0.000220068	0.000577866	0.037452129
g__Subdoligranulum	0.355975081	0.487335734	0.734307021	0.039303416	0.792398019	0.351956274	0.068646557
g__Escherichia-Shigella	0.34161808	0.544927511	0.721633683	0.363214218	0.953785778	0.558021584	0.143300329
g__Megamonas	0.004116274	0.001845877	0.109033365	0.125203933	0.119301572	0.827473965	0.604838968
g__Romboutsia	0.393786984	0.931453907	0.08472491	0.070394201	0.077794157	0.071150945	0.779090611
g__Bacteroides	0.74407337	0.269525734	0.642230725	0.971624377	0.62538284	0.849365391	0.522268197
g__Agathobacter	0.00906	0.04276	0.29926	0.00024	0.28788	0.47117	0.62778

	9867	7862	7792	507	3197	3287	6673
g__Anaerostipes	0.378716229	0.281352257	0.287041159	0.425039635	0.407620091	0.828586569	0.95673828
g__Ruminococcus	0.084682259	0.794034169	0.691163875	0.02412188	0.857416734	0.077384959	0.317114774
g__Klebsiella	0.412592643	0.604974892	0.943911047	0.033917912	0.985967441	0.0916882	0.216206938
g__norank_f__Eubacterium_coprostanoligenes_group	0.790035249	0.932260683	0.003397182	0.020951121	0.001530014	4.41E-06	0.040157591
g__Dorea	0.165613505	0.023720234	0.579438618	0.080683252	0.704524107	0.081016774	0.019967483

Original blots presented in the manuscript

Fig 8b IL-1β

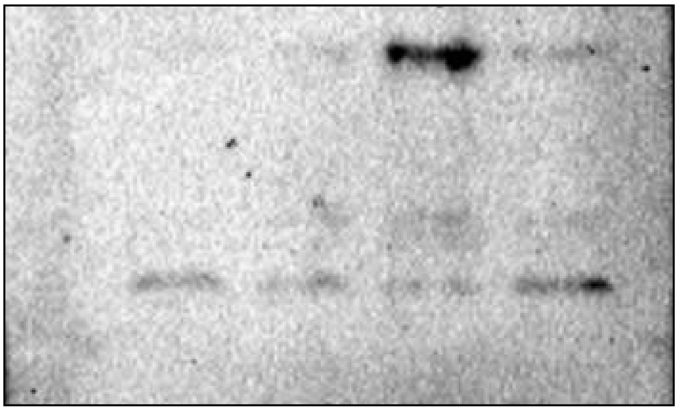

Fig 8b TNF-α

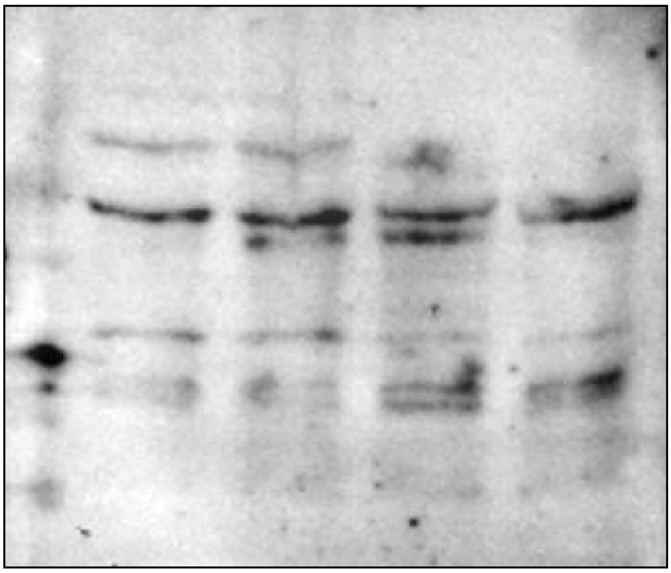

Fig 8b NF-κB

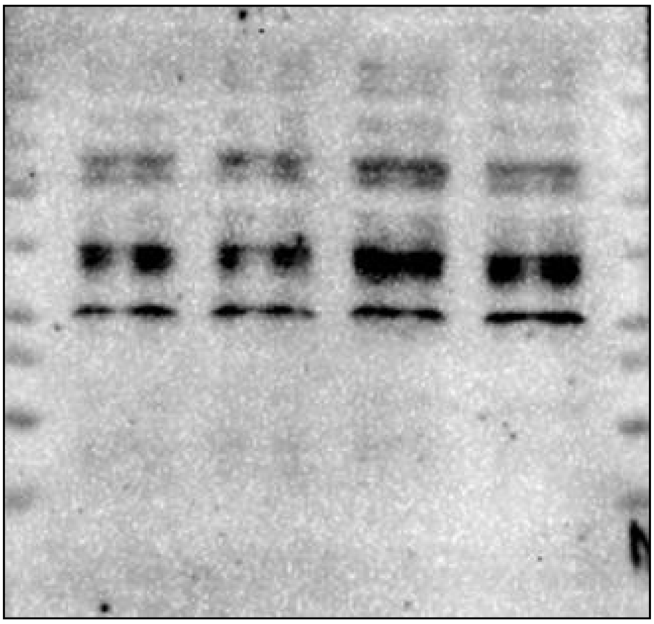

Fig 8b Actin

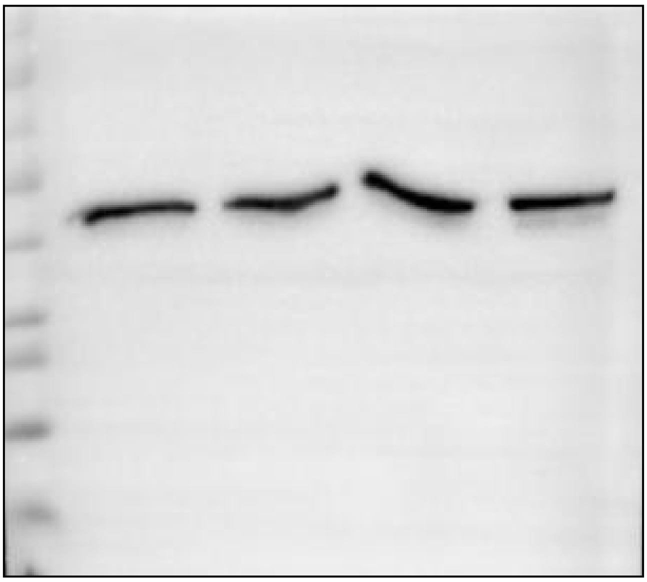

Fig 9a BDNF

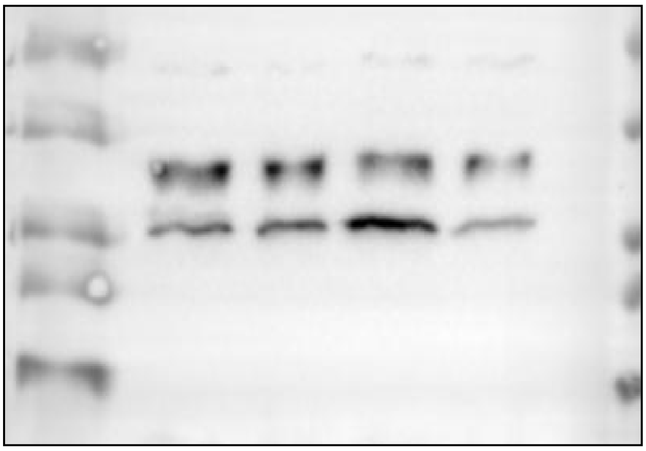

Fig 9a NGF

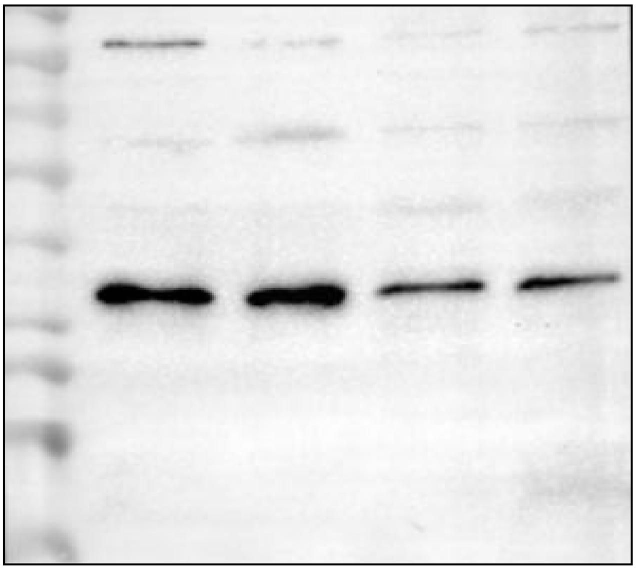

Fig 9a NT-3

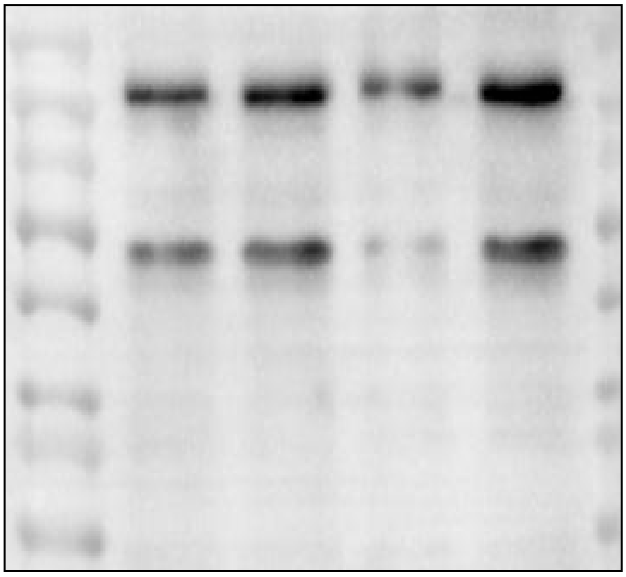

Fig 9a Actin

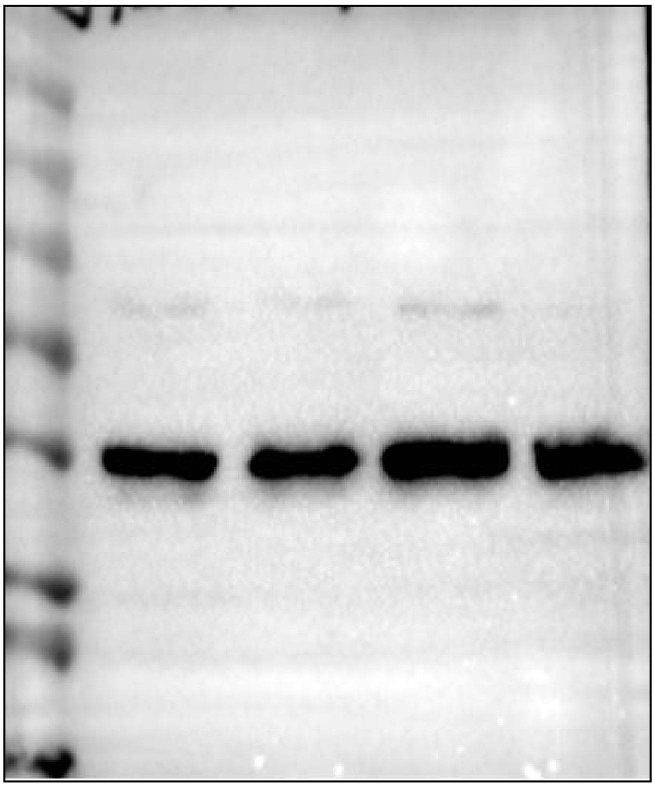

Supplement: Supplementary file 1 — Supplementary material [file 41522_2023_466_MOESM1_ESM.pdf]
