# Supplementary material for: Spinal cord injury-induced gut dysbiosis influences neurological recovery partly through short-chain fatty acids
Source: NPJ Biofilms Microbiomes. 2023 Dec 14;9:99. doi: 10.1038/s41522-023-00466-5 (PMC10719379; doi:10.1038/s41522-023-00466-5)
Supplement: Supplementary file 2 — Reporting Summary [file 41522_2023_466_MOESM2_ESM.pdf]

nature portfolio

Corresponding author(s): Zhiguo Chen
Last updated by author(s): Nov 21, 2023

Reporting Summary

Nature Portfolio wishes to improve the reproducibility of the work that we publish. This form provides structure for consistency and transparency in reporting. For further information on Nature Portfolio policies, see our Editorial Policies and the Editorial Policy Checklist.

Statistics

For all statistical analyses, confirm that the following items are present in the figure legend, table legend, main text, or Methods section.

n/a	Confirmed	
☐	☒	The exact sample size (n) for each experimental group/condition, given as a discrete number and unit of measurement
☐	☒	A statement on whether measurements were taken from distinct samples or whether the same sample was measured repeatedly
☐	☒	The statistical test(s) used AND whether they are one- or two-sided<br>*Only common tests should be described solely by name; describe more complex techniques in the Methods section.*
☒	☐	A description of all covariates tested
☐	☒	A description of any assumptions or corrections, such as tests of normality and adjustment for multiple comparisons
☐	☒	A full description of the statistical parameters including central tendency (e.g. means) or other basic estimates (e.g. regression coefficient) AND variation (e.g. standard deviation) or associated estimates of uncertainty (e.g. confidence intervals)
☐	☒	For null hypothesis testing, the test statistic (e.g. F, t, r) with confidence intervals, effect sizes, degrees of freedom and P value noted<br>*Give P values as exact values whenever suitable.*
☒	☐	For Bayesian analysis, information on the choice of priors and Markov chain Monte Carlo settings
☒	☐	For hierarchical and complex designs, identification of the appropriate level for tests and full reporting of outcomes
☒	☐	Estimates of effect sizes (e.g. Cohen's d, Pearson's r), indicating how they were calculated

Our web collection on statistics for biologists contains articles on many of the points above.

Software and code

Policy information about availability of computer code

Data collection	16SrRNA sequencing is based on the Illumina MiSeq platform (2 × 300 bp) (Illumina, San Diego,USA). SCFAs were examined with the gas chromatography–mass spectrometry (GC-MS/MS 7890B7000D; Agilent Technologies Inc.) using the silica capillary column (DB-FFAP, 30 m × 0.25 mm × 0.25 µm, Agilent J&W)., Western Blot image was acquired by ChemiDoc MP System (Bio-Rad, Hercules, CA, USA); Immunohistochemistry image was acquired either by a Nikon Eclipse Ti series fluorescencet microscope using the NIS-Elements AR software(Nikon, NY, USA) or scaned by a TissueFAX (TissueGnostics, Vienna, Austria) slide scanner; open field test was recorded by TopScan software (Clever Systems, Reston, VA)
Data analysis	Sequence analysis was performed using QIIME2 (version 2020.2) with additional analyses performed in R version 3.3.1. Mass spectrometry data was processing Using MassHunter Quantitative Analysis 8.0.598.0(Agilent Technologies, United States). Western Blot image was analyzed by Quantity One (Bio-Rad, Hercules, CA, USA); Immunohistochemistry image was analyzed by Image Pro Plus7.0 (Media Cybernetics, Silver Spring, MD, USA) or ImageJ (Bethesda, Maryland, USA); open field test was analyzed by TopScan software (Clever Systems, Reston, VA)

For manuscripts utilizing custom algorithms or software that are central to the research but not yet described in published literature, software must be made available to editors and reviewers. We strongly encourage code deposition in a community repository (e.g. GitHub). See the Nature Portfolio guidelines for submitting code & software for further information.

Data

Policy information about availability of data

All manuscripts must include a data availability statement. This statement should provide the following information, where applicable:

- Accession codes, unique identifiers, or web links for publicly available datasets
- A description of any restrictions on data availability
- For clinical datasets or third party data, please ensure that the statement adheres to our policy

All data supporting the findings of this study are available within the paper, its supplementary Information source data files, and deposited data (see data availability statements).

Research involving human participants, their data, or biological material

Policy information about studies with human participants or human data. See also policy information about sex, gender (identity/presentation), and sexual orientation and race, ethnicity and racism.

Reporting on sex and gender	Individuals of both sexes were enrolled in this study. No analysis between sexes was performed. The study design did not take into account either sex or gender. Patient enrollment followed a "first come, first served" approach. Sex and gender identification was self-reported.
Reporting on race, ethnicity, or other socially relevant groupings	The study design did not take into account any socially constructed or socially relevant catregorizations. Patient enrollment followed a "first come, first served" approach.
Population characteristics	Patient inclusion criteria were (1) SCI of neurological completeness [American Spinal Injury Association (ASIA) grade A], (2) patients aged 18–60 years, and (3) traumatic SCI. The exclusion criteria were (1) those had type 1, 2, 6, 7 stools according to Bristol Stool Form Scale, (2) Using any pharmacological and Chinese traditional agents for improving evacuations, (3) prebiotic, probiotic, antibiotic or bowel preparation use within 2 weeks prior to commencing the study, (4) SCI of incompleteness, and (5) metabolic diseases, gastrointestinal diseases, multiple sclerosis, and immune diseases. Before enrollment, all participants underwent general and full ASIA examinations. Meanwhile, normal subjects were also enrolled based on the following criteria: (1) those aged 18–60 years and (2) without a history of metabolic diseases, immune disease, multiple sclerosis, or gastrointestinal diseases. Each participant was enrolled before sample collection, and informed consent was provided after being fully informed of the sampling procedure and research options.
Recruitment	Patients were enrolled in the clinical trial following a "first come, first served" principle, in accordance with the eligibility criteria. Informed consent was obtained from all participants. We do not identify any bias that could have impacted the results.
Ethics oversight	The trail was approved by the Ethics Committee of China Rehabilitation Research Center and all participants provided written informed consent

Note that full information on the approval of the study protocol must also be provided in the manuscript.

Field-specific reporting

Please select the one below that is the best fit for your research. If you are not sure, read the appropriate sections before making your selection.

☒ Life sciences ☐ Behavioural & social sciences ☐ Ecological, evolutionary & environmental sciences

For a reference copy of the document with all sections, see nature.com/documents/nr-reporting-summary-flat.pdf

Life sciences study design

All studies must disclose on these points even when the disclosure is negative.

Sample size	No statistical methods were used to pre-determine sample sizes, but our sample sizes are similar to those in previous publications (PMID: 37308588,PMID: 35467388,PMID: 37419902). All animal, sample, and biological replicate numbers in this study align with well-accepted standards from the literature for each method.
Data exclusions	No results were excluded except in cases of animal death
Replication	The number of repetitions (individual data points from separate animals) is indicated in figures and legends. For assays such as western blotting, immunohistochemical analysis, and SCFA analysis, we conducted at least three experimental replicates for each assay. All attempted replications were successful.
Randomization	Individual samples/animals were randomized through numbering.
Blinding	All data analysis was conducted blindly with respect to experimental conditions.

Behavioural & social sciences study design

All studies must disclose on these points even when the disclosure is negative.

Study description

Research sample

Sampling strategy

Data collection

Timing

Data exclusions

Non-participation

Randomization

Ecological, evolutionary & environmental sciences study design

All studies must disclose on these points even when the disclosure is negative.

Study description

Research sample

Sampling strategy

Data collection

Timing and spatial scale

Data exclusions

Reproducibility

Randomization

Blinding

Did the study involve field work? ☐ Yes ☐ No

Field work, collection and transport

Field conditions

Location

Access & import/export

Disturbance

Reporting for specific materials, systems and methods

We require information from authors about some types of materials, experimental systems and methods used in many studies. Here, indicate whether each material, system or method listed is relevant to your study. If you are not sure if a list item applies to your research, read the appropriate section before selecting a response.

Materials & experimental systems

n/a	Involved in the study
☐	☒ Antibodies
☒	☐ Eukaryotic cell lines
☒	☐ Palaeontology and archaeology
☐	☒ Animals and other organisms
☐	☒ Clinical data
☒	☐ Dual use research of concern
☒	☐ Plants

Methods

n/a	Involved in the study
☒	☐ ChIP-seq
☒	☐ Flow cytometry
☒	☐ MRI-based neuroimaging

Antibodies

Antibodies used

Primary antibodies used in this study:
Rabbit anti-GFAP (1:500)(sigama)(Cat# HPA056030)(Lot# A104581)
Mouse anti-NF(1:200)(Cell Signaling)(Cat# 2836)(Lot# 4)
Rabbit anti-NeuN(1:100)(Abcam)(Cat# ab177487)(Lot# GR249899-26)
Rabbit anti-Synapsin(1:100)(Abcam)(Cat# ab64581)(Lot# GR3263820-2)
Rabbit anti-Iba-1(1:100)(GeneTex)(Cat# GTX100042)(Lot# No.41556)
Rabbit anti-BDNF(1:500)(Abcam)(Cat# ab108319)(Lot# GR3227037-16)
Rabbit anti-NGF(1:500)(Abcam)(Cat# ab52918)(Lot# GR226375-45)
Rabbit anti-NT-3(1:500)(Abcam)(Cat# ab16640)(Lot# GR3285318-2)
Rabbit anti-IL-1β(1:100)(Abcam)(Cat# ab9722)(Lot# GR309542-23)
Rabbit anti-TNF-α(1:100)(Abcam)(Cat# ab6671)(Lot# 3214221-16)
Rabbit anti-β-actin(1:100)(Abcam)(Cat# ab8227)(Lot# GR206472-1)
Secondary antibodies used in this study:
Donkey anti-Rabbit IgG (H+L) Secondary Antibody, Alexa Fluor 448 (1:500) (Invitrogen, OR) (Cat # A-21206) (Lot # 1531671)
Donkey anti-Mouse IgG (H+L) Secondary Antibody, Alexa Fluor 594 (1:500) (Invitrogen, OR) (Cat # A21203) (Lot # 1608644)
Cy3 conjugated Goat Anti-Rabbit IgG (H+L)Secondary Antibody,Servicebio (1:500) (Cat # GB21303) (Lot # 194308)
HRP-labeled Goat Anti-Rabbit IgG(H+L) Secondary Antibody,Servicebio (1:1000) (Cat # GB23303) (Lot # 192941)

Validation

All antibodies were validated by the manufacturer or used in published research articles.
Rabbit anti-GFAP (1:500)(sigama)(Manufacturer website states this antibody has been validated with human and mouse, validate for immunoblotting, immunofluorescence and immunohistochemistry).
Mouse anti-NF(1:200)(Cell Signaling) (Manufacturer website states this antibody has been validated with human, mouse and rat; Validate for immunofluorescence and immunohistochemistry and Western blot).
Rabbit anti-NeuN(1:100)(Abcam)(Manufacturer website states this antibody has been validated with mouse primary neuron cells and human neurons, human iPSC-Derived glutamatergic neurons, human neuroblastoma cell line from bone marrow by Immunofluorescence).
Rabbit anti-Synapsin(1:100)(Abcam)(PMID: 33678185)(Manufacturer website states this antibody reacts with: mouse, rat, goat; Validate for Western blot, Immunoprecipitation and Immunofluorescence).
Rabbit anti-Iba-1(1:100)(GeneTex)(Manufacturer website states this antibody reacts with: human, mouse, rat; tested for the following application: Western blot, Immunocytochemistry/Immunofluorescence, Immunohistochemistry, and fluorescence-activated cell sorting)
Rabbit anti-BDNF(1:500)(Abcam)(Manufacturer website states this antibody has been validated with mouse, rat and human hippocampus lysate by Western blot).
Rabbit anti-NGF(1:500)(Abcam)(Manufacturer website states This antibody has been validated with mouse, rat and human fetal brain lysate, fetal thymus lysate, thyroid lysate and HeLa cell lysate by Western blot).
Rabbit anti-NT-3(1:500)(Abcam)(Manufacturer website states this antibody has been validated with mouse, rat and human brain tissue lysate by Western blot).
Rabbit anti-IL-1β(1:100)(Abcam)(Manufacturer website states this antibody has been validated in mouse by western blot)
Rabbit anti-TNF-α(1:100)(Abcam)(PMID:35267945)(Manufacturer website states This antibody Reacts with: human, cynomolgus monkey, mouse, recombinant fragment?; Validate for Western blot, Immunocytochemistry, Immunohistochemistry, ELISA and Neutralising).
Rabbit anti-β-actin(1:100)(Abcam)(Manufacturer website states this antibody reacts with: mouse, rat, rabbit, chicken, cow, dog, human, xenopus laevis, fish, Chinese hamster; Validate for Western blot, Immunocytochemistry, Immunohistochemistry.)

Eukaryotic cell lines

Policy information about cell lines and Sex and Gender in Research

Cell line source(s)

Authentication

Mycoplasma contamination

Commonly misidentified lines
(See ICLAC register)

Palaeontology and Archaeology

Specimen provenance	
Specimen deposition	
Dating methods	

☐ Tick this box to confirm that the raw and calibrated dates are available in the paper or in Supplementary Information.

Ethics oversight	

Note that full information on the approval of the study protocol must also be provided in the manuscript.

Animals and other research organisms

Policy information about studies involving animals; ARRIVE guidelines recommended for reporting animal research, and Sex and Gender in Research

Laboratory animals	Adult female C57BL/6N mice weighing 18–22 g were used.
Wild animals	No wild animal was used in this study.
Reporting on sex	Due to the relative ease of bladder expression and lower risk of bladder infections and other complications (e.g., urethral blockages) in female mice compared to males (PMID: 32142803), only female mice were used in the current study.
Field-collected samples	No field-collected sample used in this study
Ethics oversight	Animal care and the experimental protocols were approved by the The Animal Care and Use Committee of Capital Medical University

Note that full information on the approval of the study protocol must also be provided in the manuscript.

Clinical data

Policy information about clinical studies

All manuscripts should comply with the ICMJE guidelines for publication of clinical research and a completed CONSORT checklist must be included with all submissions.

Clinical trial registration	ChiCTR-RPC-17010621
Study protocol	The clinical protocol is included
Data collection	Data was collected from eligible patients recruited between from December 2020 to March 2021 in China. The cut-off date for data analysis was July 19th, 2021
Outcomes	The primary objective was to evaluate the change of intestinal microbiota between SCI patients and normal objective. Secondary objective was to evaluate the change of Metabolites of intestinal microbiota between SCI patients and normal objective.

Dual use research of concern

Policy information about dual use research of concern

Hazards

Could the accidental, deliberate or reckless misuse of agents or technologies generated in the work, or the application of information presented in the manuscript, pose a threat to:

No	Yes	
☐	☐	Public health
☐	☐	National security
☐	☐	Crops and/or livestock
☐	☐	Ecosystems
☐	☐	Any other significant area

Experiments of concern

Does the work involve any of these experiments of concern:

No	Yes	
☐	☐	Demonstrate how to render a vaccine ineffective
☐	☐	Confer resistance to therapeutically useful antibiotics or antiviral agents
☐	☐	Enhance the virulence of a pathogen or render a nonpathogen virulent
☐	☐	Increase transmissibility of a pathogen
☐	☐	Alter the host range of a pathogen
☐	☐	Enable evasion of diagnostic/detection modalities
☐	☐	Enable the weaponization of a biological agent or toxin
☐	☐	Any other potentially harmful combination of experiments and agents

Plants

Seed stocks

Novel plant genotypes

Authentication

ChIP-seq

Data deposition

☐ Confirm that both raw and final processed data have been deposited in a public database such as GEO.

☐ Confirm that you have deposited or provided access to graph files (e.g. BED files) for the called peaks.

Data access links
May remain private before publication.

Files in database submission

Genome browser session
(e.g. UCSC)

Methodology

Replicates

Sequencing depth

Antibodies

Peak calling parameters

Data quality

Software

Flow Cytometry

Plots

Confirm that:

☐ The axis labels state the marker and fluorochrome used (e.g. CD4-FITC).

☐ The axis scales are clearly visible. Include numbers along axes only for bottom left plot of group (a 'group' is an analysis of identical markers).

☐ All plots are contour plots with outliers or pseudocolor plots.

☐ A numerical value for number of cells or percentage (with statistics) is provided.

Methodology

Sample preparation

Instrument

Software

Cell population abundance

Gating strategy

☐ Tick this box to confirm that a figure exemplifying the gating strategy is provided in the Supplementary Information.

Magnetic resonance imaging

Experimental design

Design type

Design specifications

Behavioral performance measures

Acquisition

Imaging type(s)

Field strength

Sequence & imaging parameters

Area of acquisition

Diffusion MRI ☐ Used ☐ Not used

Preprocessing

Preprocessing software

Normalization

Normalization template

Noise and artifact removal

Volume censoring

Statistical modeling & inference

Model type and settings

Effect(s) tested

Specify type of analysis: ☐ Whole brain ☐ ROI-based ☐ Both

Statistic type for inference

(See Eklund et al. 2016)

Correction

Models & analysis

n/a	Involved in the study
☐	☐ Functional and/or effective connectivity
☐	☐ Graph analysis
☐	☐ Multivariate modeling or predictive analysis

Functional and/or effective connectivity

Graph analysis

Multivariate modeling and predictive analysis
